# Efficacy of Danshen Class Injection in the Treatment of Acute Cerebral Infarction: A Bayesian Network Meta-Analysis of Randomized Controlled Trials

**DOI:** 10.1155/2019/5814749

**Published:** 2019-02-03

**Authors:** Shi Liu, Kaihuan Wang, Xiaojiao Duan, Jiarui Wu, Dan Zhang, Xinkui Liu, Yi Zhao

**Affiliations:** Department of Clinical Chinese Pharmacy, School of Chinese Materia Medica, Beijing University of Chinese Medicine, Beijing 100029, China

## Abstract

**Introduction:**

As a common chronic disease with high morbidity and recurrent rate, acute cerebral infarction (ACI) affects the life quality of patients and gives them heavy psychological burden. And Danshen class injections (DSCIs) are commonly adopted in treating ACI. So, this network meta-analysis (NMA) was designed to assess the clinical efficacy of eight kinds of DSCIs.

**Methods:**

A systematic literature search was performed in PubMed, the Cochrane Library, Embase, the China National Knowledge Infrastructure Database, the China Biomedical Literature Service System, the Chinese Scientific Journals Full-text Database, and Wanfang database from their inception to 16^th^ Dec. 2017 aiming to collect related randomized controlled trials (RCTs). And then data were analyzed through Stata and WinBUGS software based on the Bayesian statistical model. The results were reported as odds ratio (OR) and mean difference (MD) with 95% credible intervals (CIs). The consistency test between direct and indirect comparisons was also evaluated and inconsistency factor was presented to manifest the heterogeneity among them. Meanwhile, the surface under the cumulative ranking probabilities (SUCRA) was utilized to rank the treatments in different outcomes.

**Results:**

Finally, 157 RCTs with 15570 patients were included. A total of eight types DSCIs were identified. Based on the results, Sodium Tanshinone IIA Sulfonate injection (STS) plus western medicine (WM) had better effect on the clinical effectiveness rate, neurological impairment, and activities of daily living function than others. Meanwhile, Danhong injection (DS) and Danshen Salvianolic Acids injection (DSSA) had excellent performance in perfecting hemorheological indexes.

**Conclusions:**

In conclusion, STS plus WM may be the optimum treatment for ACI. The following therapies were DS plus WM and DSSA plus WM. Nevertheless, in terms of the limitations of the study, more large samples, multicenter, and double-blind RCTs are still needed for validating our findings.

## 1. Introduction

Acute cerebral infarction (ACI), a common cerebral vascular disease, refers to brain dysfunction caused by ischemia and anoxia of the brain tissue [1]. If a patient with ACI gets an ischemic stroke, his neurological deficiency will deteriorate stepwise [2]. Globally, it is estimated that approximately 17 million patients suffer from ACI per year, and ACI is a recurrent disease as well due to its long-term rehabilitation [3]. As a common chronic disease with high morbidity and recurrent rate, ACI affects the life quality of patients and gives them heavy psychological burden [3]. The therapeutic principle is to boost cerebral blood flow, protect brain tissue, and promote brain metabolism. However, some stroke patients have no access to appropriate management because of the lack of available experts (especially in nonurban areas), financial factors, healthcare knowledge, and delayed hospital admission. And most of them are unable to access a hospital with the appropriate facilities within the 4.5-hour treatment window [4, 5]. And standardization of stroke unit care has not been popularized in China, and there is still a lack of unified models and standards for the construction and promotion of stroke unit care [6]. Therefore, it is necessary to seek other promising therapeutic approaches to manage ACI. The lack of effective and widely applicable pharmacological treatments for patients with ACI has led to a growing interest in traditional Chinese medicine [7].

In traditional Chinese medicine theory, ACI is defined as apoplexy and its pathogenesis is the disorder of blood and* Qi* [1, 8]. Thus, the main therapeutic principle is to invigorate the blood circulation and remove blood stasis. Danshen, as a representative Chinese herbal medicine, is widely used to promote blood circulation and remove stagnation of blood. Besides, the relative pharmacological experiments demonstrated that Danshen possesses the characteristics of improving mini-circulation, preventing thrombus, and improving neurological function. Tanshinone, salvianolic acid, and polysaccharides are main ingredients in Danshen [9]. In this context, the Chinese herbal injections (CHIs) that contain the extractive from Danshen are commonly adopted in treating ACI due to remarkable curative and quick efficacy [10–16]. We deemed them as Danshen class injections (DSCIs), namely, Danhong injection (DH), Danshen injection (DS), Danshenchuanxiongqin injection (DSCXQ), Danshen Salvianolic Acids injection (DSSA), Fufang Danshen injection (FFDS), Guanxinning injection (GXN), Salvianolate injection (SI), and Sodium Tanshinone IIA Sulfonate injection(STS). All of them were approved by China Food and Drug Administration on the market of ACI.

Existing systemic reviews demonstrated that DSCIs have obtained the desired effects in treating ACI based on the western medicine (WM), while clinical trials directly comparing between DSCIs are insufficient, and a comprehensive recognition of DSCIs on ACI remains unknown as a result. Therefore, it is necessary to conduct a NMA to figure out the comparative efficacy among the different DSCIs. Compared with the conventional pairwise meta-analysis, NMA can rank the multiple interventions via indirect and direct comparison analysis. Meanwhile, the effects of interventions based on the probability of optimal treatment can be obtained [17, 18]. The goal of this study was to sort out the efficacy between DSCIs with NMA and provide more insights for selection of ACI treatment.

## 2. Methods

A completed PRISMA checklist was included as additional file (Table S1).

### 2.1. Inclusion Criteria

#### 2.1.1. Types of Studies

Randomized controlled trials (RCTs) of DSCIs in the treatment of ACI with WM, regardless of using blind method.

#### 2.1.2. Types of Participants

All patients were diagnosed with ACI according to cranial computed tomography and/or magnetic resonance imaging. In accordance with “the Chinese guidelines for diagnosis and treatment for acute ischemic stroke 2014” [19], the acute phase of ACI generally referred to 2 weeks after the onset of disease. Thus, this NMA only enrolled patients with the course of disease within 2 weeks. There is no limitation on age, gender, ethnicity, and disease severity.

#### 2.1.3. Types of Interventions

This NMA involved the RCTs with DSCI+WM vs WM or DSCI+WM vs DSCI+WM. WM contained the way of dehydration, lowering intracranial pressure, anticoagulation, antiplatelet aggregation, and improving brain metabolism, such as aspirin, anticoagulants, dehydrant, and neuroprotectant. There is no limitation on the dosage and course of treatment. If there were other complications, the corresponding treatments should be taken.

#### 2.1.4. Types of Outcomes

Main outcome measures were clinical effectiveness rate and neurological impairment. Clinical effectiveness rate and neurological impairment were calculated in accord with National Institute of Health Stroke Scale: neurologic deficit scores reduced from 100% to 18% were deemed as the class of recovery, and those decreased by 0%-17% or under 0 were regarded as invalidation or deterioration. The clinical effectiveness rate was calculated by this formula: (number of recovery patients / total number of patients) *∗* 100%. Activities of daily living function, hemorheological indexes (whole blood high shear viscosity, whole blood low shear viscosity, plasma viscosity, and fibrinogen), and adverse drug reactions/adverse drug events (ADRs/ADEs) were defined as the secondary outcomes.

This NMA excluded the RCTs with following conditions: (1) Chinese herbal medicine, acupuncture, and other traditional Chinese medicine treatment methods were received in trials; (2) data of interventions were incorrect or incomplete; (3) articles was unavailable for the full text; (4) reports are duplicated.

### 2.2. Literature Search

An electronic search of the PubMed, the Cochrane Library, Embase, the China National Knowledge Infrastructure Database, the China Biomedical Literature Service System, the Chinese Scientific Journals Full-text Database, and Wanfang database was performed for RCTs focusing on DSCIs against ACI. The MeSH terms and free-text words were utilized to retrieve articles. The search words mainly included “brain infarction”, “cerebral infarction”, “stoke”, “brain embolism”, “ischemic stroke”, “cerebrovascular disorders”, “danhong injection”, “danshen injection”, “salvianolate injection”, “danshenchuanxiongqin”, “salvia ligustrazine injection”, “danshen salvianolic acids injection”, “fufang danshen injection”, “compound danshen injection”, “composite salvia miltiorrhiza injection”, “guanxinning injection”, “salvia miltiorrhiza injection”, and “tanshinone IIA injection”. The specific retrieval strategy is shown in Table S2.

The ethical approval was not necessary in this NMA because it just gathered the RCTs from literature search. This procedure did not involve any patients' personal data.

### 2.3. Study Selection and Data Extraction

Two reviewers independently read the titles and abstracts of literature to filter out the unrelated articles, reviews, and pharmacological experiments. For the potential articles, reviewers read the full text to determine whether it met the inclusion criteria or not. If there were any difference in the study selection, a third investigator participated to resolve the disagreement.

The following factors of each embedded RCTs were recorded: (1) essential information of included studies (the name of first author and year of publication); (2) patient characteristics (sample size, gender, age, intervention in detail, course of treatment); (3) measurements of outcomes; (4) details of trial design and key factors of bias.

### 2.4. Quality Evaluation

The methodological quality of the involved RCTs was evaluated by using the modified Jadad scale. Random sequence generation, allocation concealment, blinding, discharge of patients were assessed. When random sequence generation, allocation concealment, and blinding were appropriate, the RCTs were scored 2 points, 1 point when the information was unclear, and 0 point when inappropriate. If discharge of patients was described, the score was 1 point; otherwise, the score was 0. If a RCT got score 7, it would be evaluated as perfect study, 4-7 belonging to high quality, 1-3 deemed as low quality. A crosscheck was conducted after evaluation. Disagreements were solved by discussion or arbitrated by third reviewer.

### 2.5. Statistical Analysis

WinBUGS 1.4.3 software was utilized to analyze data by a random-effects model through Markov chain Monte Carlo methods. Each chain set 50 000 iterations, and the first 20 000 were used for “burn-in” in order to eliminate the impact of initial value. The remaining 30 000 were applied for the posterior summaries. The odds ratio (OR) and mean difference (MD) with 95% credible intervals (95% CIs) were estimated for dichotomous and continuous data, respectively. When the 95% CIs of OR did not contain 1 and 95% CIs of MD did not cover 0, it would be regarded as the statistically significant difference between groups [20]. The SUCRA of each outcome was calculated to predict the rank of interventions [21].

Graphical representation of results, consistency test, and publication bias test were performed by Stata 13.0 software. In the network graph, nodes represented interventions, and link lines indicated evidence of direct comparison between two interventions. The size of each node was proportional to the enrolled RCTs. The widths of each link represented the cumulative number of RCTs for each comparison. To evaluate the consistency of each closed loop, the inconsistency factors (IF) and their 95% CIs were calculated. When the lower bound of 95% CIs was equal to 0, it was regarded as a better consistency [22]. A comparison-adjusted funnel plot was used to assess publication bias [23]. If funnel plot was symmetrical, there was no obvious publication bias.

## 3. Results

### 3.1. Literature Search

**Figure 1:**
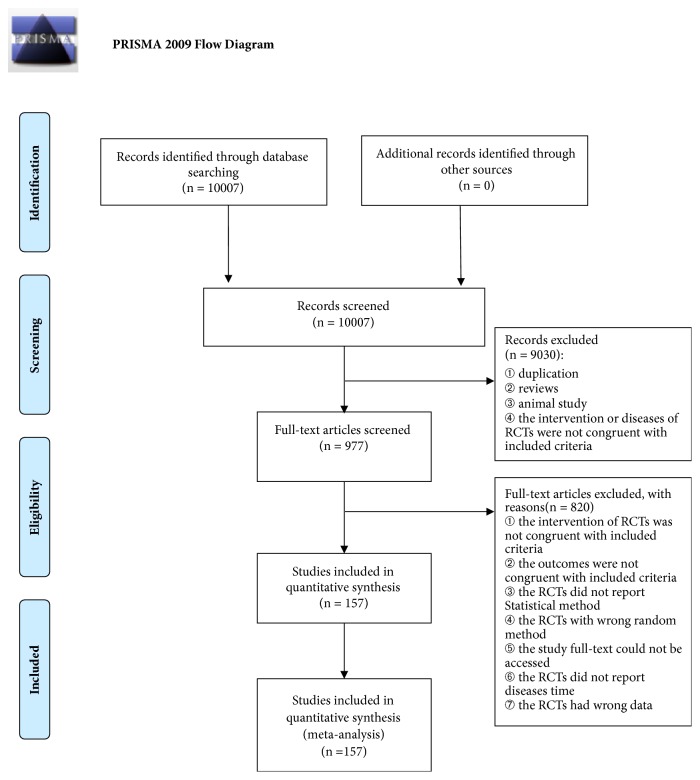
Flow chart of the search for eligible studies.

As shown in Figure 1, a total of 10,007 citations were initially detected by the search strategy, and 157 articles were qualitatively analyzed and included in the current NMA ultimately. They were all published in China from 2005 to 2016.

### 3.2. Characteristics of Included Studies

**Figure 2:**
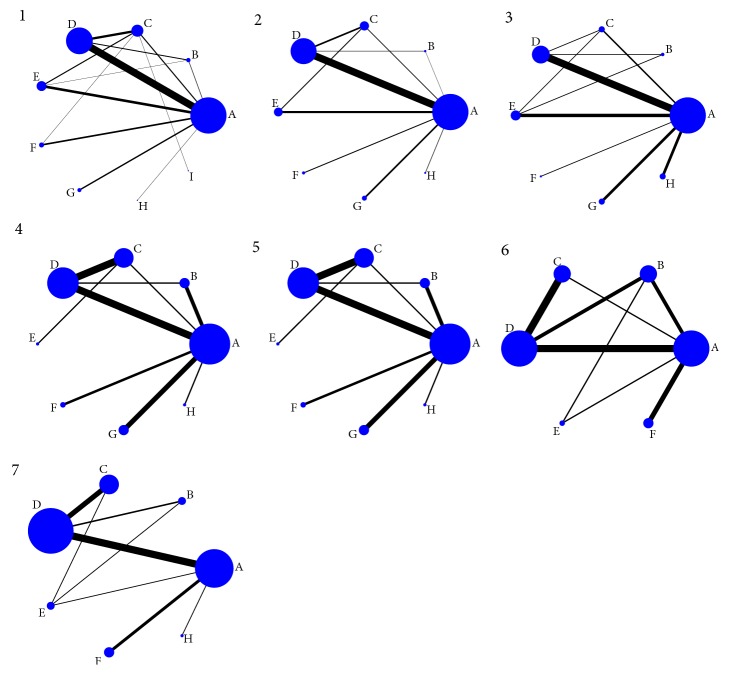
Network graph for outcomes (1: clinical effectiveness rate; 2: neurological impairment; 3: activities of daily living function; 4: whole blood high shear viscosity; 5: whole blood low shear viscosity; 6: plasma viscosity; 7: fibrinogen; A: WM; B: Danshen injection + WM; C: Fufang Danshen injection + WM; D: Danhong injection + WM; E: Danshenchuanxiongqin injection + WM; F: Sodium Tanshinone IIA Sulfonate injection + WM; G: Salvianolate injection + WM; H: Danshen Salvianolic Acids injection + WM; I: Guanxinning injection + WM).

A total of 157 RCTs involving 15, 570 patients were eligible. The experiment group enrolled 7, 875 cases and the control group contained 7, 695 patients with ACI, and their age was ranged from 32 to 96. The number of male patients (58.1%) predominated compared to this of women. All the RCTs reported no significant difference in age, gender, course, and severity of disease between the two groups. Moreover, the sample size ranged from 40 to 300. All the embedded RCTs were two-arm studies. This NMA included 9 interventions, namely WM, DH+WM, DS+WM, DSCXQ+WM, DSSA+WM, FFDS+WM, GXN+WM, SI+WM, and STS+WM. Details were shown in Table S3. A network diagram of all embedded studies was shown in Figure 2.

### 3.3. Quality of the Included Studies

Of the 157 included RCTs, 29 RCTs produced stochastic sequences by table of random number, 11 referred to the blinding, and 2 trials reported the incomplete outcomes. None of the RCTs mentioned the information of allocation concealment. And all RCTs were without drop-out cases. The modified Jadad scale was used to evaluate the methodological quality of included RCTs. The results were listed as follows: 1 point: 2 RCTs (2/157, 1.27%), 2 points: 117 RCTs (117/157, 74.5%), 3 points: 9 RCTs (9/157, 5.73%), 4 points: 27 RCTs (27/157, 17.2%), and 5 points: 2 RCTs (2/157, 1.27%). Details were presented in Table S3.

### 3.4. Clinical Effectiveness Rate

The 133 RCTs referred to the clinical effectiveness rate, involving 9 interventions. For clinical effectiveness rate, when compared with WM, all treatments, including DH+WM (OR=4.03, 95% CIs: 3.41~4.69), DS+WM (OR=1.64, 95% CIs: 1.09~2.55), DSCXQ+WM (OR=3.61, 95% CIs: 2.80~4.61), DSSA+WM (OR=5.52, 95% CIs: 1.37~17.00), FFDS+WM (OR=1.40, 95% CIs: 1.11~1.74), GXN+WM (OR=922.30, 95% CIs: 2.05~424.80), SI+WM (OR=3.15, 95% CIs: 2.35~4.23), and STS+WM (OR=4.15, 95% CIs: 2.84~5.87) showed significant clinical efficacy. DH+WM (OR=2.56, 95% CIs: 1.65~3.70), DSCXQ+WM (OR=2.30, 95% CIs: 1.40~3.46), GXN+WM (OR=672.30, 95% CIs: 1.27~271.2), SI+WM (OR=2.02, 95% CIs: 1.12~3.19), and STS+WM (OR=2.65, 95% CIs: 1.4~4.31) were more efficacious than DS+WM. In addition, DH+WM (OR=2.90, 95% CIs: 2.31~3.59), DSCXQ+WM (OR=2.60, 95% CIs: 1.95~3.48), GXN+WM (OR=668.30, 95% CIs: 1.52~296.00), SI+WM (OR=2.28, 95% CIs: 1.55~3.29), and STS+WM (OR=3.00, 95% CIs: 1.93~4.43) provided significant benefits in improving clinical effectiveness rate when compared with FFDS+WM (Table 1). There was no significant difference between other interventions.

**Figure 3:**
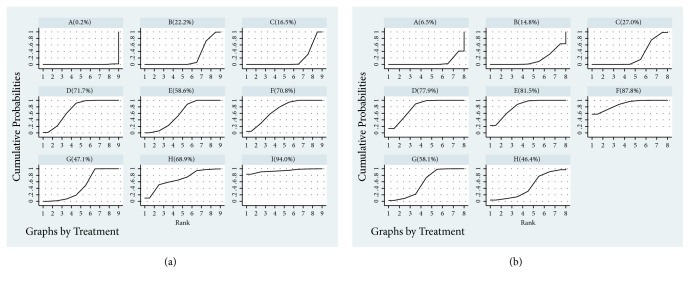
Rank of the cumulative probabilities of clinical effectiveness rate and neurological impairment ((a): clinical effectiveness rate; (b): neurological impairment; A: WM; B: Danshen injection + WM; C: Fufang Danshen injection + WM; D: Danhong injection + WM; E: Danshenchuanxiongqin injection + WM; F: Sodium Tanshinone IIA Sulfonate injection + WM; G: Salvianolate injection + WM; H: Danshen Salvianolic Acids injection + WM; I: Guanxinning injection + WM).

The SUCRA was depicted in Figure 3: GXN+WM (94.0%), DH+WM (71.7%), STS+WM (70.8%), DSSA+WM (68.9%), DSCXQ+WM (58.6%), SI+WM (47.1%), DS+WM (22.2%), and FFDS+WM (16.5%), respectively.

### 3.5. Neurological Impairment

A total of 112 trials including 8 interventions investigated neurological impairment. The results were revealed in Table 1. DH+WM (MD=-4.38, 95% CIs: -5.07~-3.70), DSCXQ+WM (MD=-4.52, 95% CIs: -5.65~-3.40), SI+WM (MD=-3.45, 95% CIs: -4.96~-1.96), and STS+WM (MD=-5.01, 95% CIs: -7.19~-2.84) could achieve a better effect in neurological impairment than WM. Simultaneously, when compared with DS+WM, DH+WM (MD=-4.01, 95% CIs: -6.40~-1.60), DSCXQ+WM (MD=-4.15, 95% CIs: -6.70~-1.60), SI+WM (MD=-3.08, 95% CIs: -5.94~-0.22), and STS+WM (MD=-4.64, 95% CIs: -7.89~-1.38) showed significant benefit in neurological impairment. And DH+WM (MD=-3.22, 95% CIs: -4.40~-2.04), DSCXQ+WM (MD=-3.37, 95% CIs: -4.78~-1.93), SI+WM (MD=-2.29, 95% CIs: -4.22~-0.35), and STS+WM (MD=-3.86, 95% CIs: -6.40~-1.36) were more effective than FFDS+WM (Table 1). No significant differences were detected during other comparisons.

The SUCRA was depicted in Figure 3: STS+WM (87.8%), DSCXQ+WM (81.5%), DH+WM (77.9%), SI+WM (58.1%), DSSA+WM (46.4%), FFDS+WM (27.0%), and DS+WM (14.8%).

### 3.6. Activities of Daily Living Function

A total of 8 interventions were included from 31 RCTs reporting activities of daily living function.  Table 2 summarized the results: compared to WM, DH+WM (MD=10.55, 95% CIs: 7.42~13.63), DSCXQ+WM (MD=9.53, 95% CIs: 5.48~13.60), DSSA+WM (MD=9.73, 95% CIs: 4.00~15.40), FFDS+WM (MD=10.83, 95% CIs: 4.68~16.87), SI+WM (MD=7.37, 95% CIs: 2.35~12.45), and STS+WM (MD=16.53, 95% CIs: 6.94~26.17) could provide significant impact on improving activities of daily living function. Meanwhile, DH+WM (MD=8.92, 95% CIs: 1.74~16.10) and STS+WM (MD=14.91, 95% CIs: 2.97~26.98) were more efficacious in activities of daily living function than DS+WM (Table 2). There was no significant difference between other interventions.

The results of SUCRA were as follows: STS+WM (92.1%), FFDS+WM (67.6%), DH+WM (67.0%), DSSA+WM (59.0%), DSCXQ+WM (57.0%), SI+WM (40.2%), DS+WM (12.5%).

### 3.7. Whole Blood High Shear Viscosity

The 24 studies involving 8 interventions referred to whole blood high shear viscosity. When compared with WM, DS+WM (MD=-1.32, 95% CIs: -2.37~-0.27), DH+WM (MD=-1.55, 95% CIs: -2.37~-0.74), and STS+WM (MD=-1.66, 95% CIs: -3.11~-0.21) were associated with lower whole blood high shear viscosity than WM alone. And DH+WM compared with FFDS+WM (MD=-1.13, 95% CIs: -1.95~-0.33) could effectively improve the whole blood high shear viscosity (Table 2). The difference between the other comparisons was not statistically significant.

The results of SUCRA were as follows: STS+WM (76.4%), DH+WM (76.2%), DSCXQ+WM (67.5%), DS+WM (65.6%), SI+WM (40.7%), DSSA+WM (37.2%), FFDS+WM (26.9%).

### 3.8. Whole Blood Low Shear Viscosity

A total of 8 interventions reported the data of whole blood low shear viscosity from 24 RCTs. As shown in Table 3, STS+WM (MD=-19.89, 95% CIs: -26.42~-13.4) could effectively improve whole blood low shear viscosity more than WM, and the difference was statistically significant. Moreover, there was a trend that STS+WM was more efficacious than DH+WM (MD=-18.55, 95% CIs: -25.93~-11.24), DS+WM (MD=-18.17, 95% CIs: -26.23~-10.16), DSCXQ+WM (MD=-18.63, 95% CIs: -30.79~-6.51), DSSA+WM (MD=19.30, 95% CIs: 8.18~30.54), FFDS+WM (MD=-19.79, 95% CIs: -27.82~-11.77), and SI+WM (MD=18.55, 95% CIs: 10.54~26.64) (Table 3). There was no statistical difference between the other interventions.

In whole blood low shear viscosity, the SUCRA was arranged as follows: STS+WM (100%), DS+WM (52.5%), DH+WM (50.4%), SI+WM (48.1%), DSCXQ+WM (45.7%), DSSA+WM (40.9%), and FFDS+WM (32.5%).

### 3.9. Plasma Viscosity

The plasma viscosity analysis included data from 25 RCTs with 6 interventions. The results were shown in Table 3: DS+WM (MD=-0.34, 95% CIs: -0.67~-0.02) and DH+WM (MD=-0.53, 95% CIs: -0.79~-0.25) could achieve a more favorable effect on PV than WM. And DH+WM was better than FFDS+WM (MD=-0.52, 95% CIs: -0.81~-0.23) (Table 3). There was no statistical difference between the other comparisons.

The results of SUCRA were as follows: DH+WM (90.1%), DSCXQ+WM (67.7%), DS+WM (65.5%), SI+WM (40.6%), FFDS+WM (20.1%).

### 3.10. Fibrinogen

The analyses of fibrinogen risk included data from 27 RCTs with 8 interventions. The results indicated that the interventions of DH+WM (MD=-1.09, 95% CIs: -1.37~-0.80), STS+WM (MD=-1.13, 95% CIs: -1.95~-0.29), and DSSA+WM (MD=-1.79, 95% CIs: -3.51~-0.06) were associated with improving fibrinogen than WM alone. In addition, DH+WM were also found to be significantly superior to DS+WM (MD=-0.83, 95% CIs: -1.45~-0.22), FFDS+WM (MD=-1.10, 95% CIs: -1.43~-0.78), DSCXQ+WM (MD=-0.73, 95% CIs: -1.29~-0.18), and SI+WM (MD=-0.65, 95% CIs: -1.26~-0.03) (Table 4). No statistically significant difference was observed for other interventions.

The results of SUCRA were as follows: DSSA+WM (89.9%), DH+WM (80.7%), STS+WM (79.8%), SI+WM (47.0%), DSCXQ+WM (42.5%), DS+WM (34.0%), FFDS+WM (12.7%).

### 3.11. Safety

Of the 157 enrolled trials, 93 RCTs did not mention the information of ADRs/ADEs, and 39 studies showed no ADRs/ADEs, and 25 studies reported 70 cases of ADRs. The number and manifestations of ADRs/ADEs in each group were as follows: WM (10 cases): local cyanosis, nausea, vomiting, skin rash, subcutaneous hemorrhage, raised glutamic pyruvic transaminase; DS+WM (6 cases): skin rash, nausea, and vomiting; DH+WM (18 cases): dizziness, flushing, palpitations, skin rash, elevated serum alanine aminotransferase, elevated urea nitrogen, low-grade fever; DSCXQ+WM (15 cases): skin rash, mild headache, belching, mental excitement; STS+WM (7 cases): phlebitis, dizziness, redness and swelling of the skin on the side of the limb; SI+WM (6 cases): digestive tract reaction, skin rash, pruritus; DSSA+WM (8 cases): redness and swelling of the lips, upper gastrointestinal bleeding, liver dysfunction, hyperuricemia, epistaxis, skin itching.

### 3.12. Publication Bias

The funnel plot of clinical effectiveness rate was not quite symmetric, meaning potential publication bias in the network (Figure 5).

### 3.13. Consistency Test

**Figure 4:**
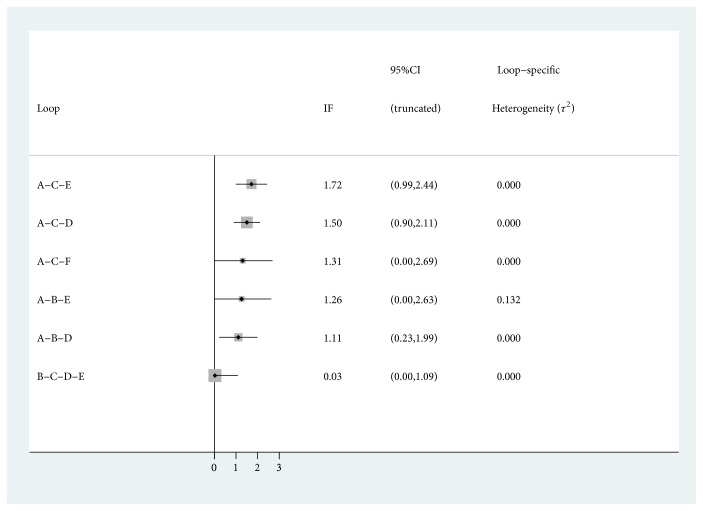
Schematic diagram of consistency.

**Figure 5:**
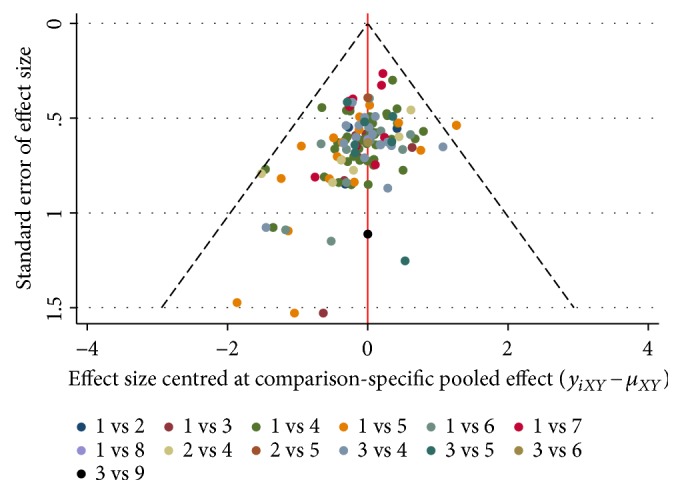
Funnel plot of performance status (1: Danshen injection + WM; 2: WM; 3: Fufang Danshen injection + WM; 4: Danhong injection + WM; 5: Danshenchuanxiongqin injection + WM; 6: Sodium Tanshinone IIA Sulfonate injection + WM; 7: Salvianolate injection + WM; 8: Danshen Salvianolic Acids injection + WM; 9: Guanxinning injection + WM).

As depicted in Figure 4, there were 6 loops, 5 of them were the three-side ring and 1 was four-side ring. The results indicated that the 95% CIs of 3 loops contained 0, and IF was between 0.03 and 1.73. Therefore, there was some inconsistency in this study.

## 4. Discussion

ACI has been a global public health issue, and the combination of DSCIs and WM was widely applied in China. Because the conventional pairwise meta-analysis comparing DSCIs directly was deficient, the comparison between multiple interventions could not be obtained. Given this, we carried out a NMA so as to access the efficacy and safety of them simultaneously.

This NMA identified 157 RCTs involving nine interventions, namely, DH+WM, DS+WM, DSCXQ+WM, DSSA+WM, FFDS+WM, GXN+WM, SI+WM, STS+WM, and WM. The data were collected from eligible RCTs in the aspects of clinical effectiveness rate, neurological impairment, activities of daily living function, hemorheological indexes, and ADRs/ADEs. To recognize patients' condition comprehensively, clinical effectiveness rate and neurological impairment situation were deemed as dominant outcomes. Besides, activities of daily living function, hemorheological indexes, and ADRs/ADEs were regarded as secondary outcomes. In terms of the primary outcomes, GXN+WM and STS+WM were superior to others. However, the efficacy of GXN could not be confirmed because only one RCT tested the efficacy of GXN plus WM. What is more, STS+WM had a noteworthy effect on increasing activities of daily living function, whole blood high shear viscosity, and whole blood low shear viscosity. Besides, DH and DSSA preformed a marked impact in reducing plasma viscosity and fibrinogen contents.

Sodium tanshinone IIA sulfonate is the main content of STS, extracted from Danshen. The previous pharmacological studies showed that STS owns a capacity of preventing central nervous system from ischemia and anoxia. It also can reduce the damage caused by ischemia reperfusion via antiapoptotic mechanism [24]. Our findings were consistent with several pairwise meta-analyses, reporting that STS exhibited a noteworthy performance on the basis of WM in improving clinical effectiveness rate of ACI patients [10]. As for DH, its effective constituents are tanshinone, safflower yellow, salvianolic acid, safflower phenolic glycosides, and so on. The results of pharmacological experiments suggested that DH had a favorable effect on improving cerebral microcirculation, nourishing brain tissue, protecting neurons, and inhibiting the inflammatory response [8]. DSSA was composed of various salvianolic acids, for instance, rosmarinic acid and alkanoic acid. As the related research demonstrated, DSSA provided benefits for protecting ischemic brain tissue and neuron. Furthermore, it also had the function of anti-inflammation [11]. Pairwise meta-analysis revealed that both of DH and DSSA could exhibit a better impact on improving clinical efficacy, neurologic deficiency, and other outcomes [11–13]. The above evidence was congruent with this NMA. And these three DSCIs had a better performance than other DSCIs in treating ACI.

Apart from efficacy, safety is another element to be considered in treatment. About 60% of RCTs did not report safety in this NMA; therefore, we could not make a conclusion of DSCIs' safety. With DSCIs widely used in treating ACI, it is necessary to monitor the ADRs/ADEs when patients received them within 30 minutes. In addition, attention should be paid to appropriate dripping speed [25].

Upon the design and contents, three merits boosted the creditability of this NMA. First of all, a comprehensive literature search and DSCIs utilization were performed in this NMA. Second, this NMA expressed the efficacy of DSCIs based on a large number of included RCTs. Meanwhile, a consistency test was applied to promote its reliability. Third, it was significant that the outcomes demonstrated patients' conditions in multiple aspects. In terms of corresponding results, this NMA offered several clinical suggestions for treatment in ACI.

Though this NMA evaluated the efficacy of DSCIs against ACI for the first time, it also had several limitations. First, the enrolled RCTs in current NMA were all published in Chinese and these trials were conducted among Chinese populations; accordingly, the efficacy of DSCIs against ACI in non-Chinese populations was still uncertain. Second, few eligible RCTs reported the outcomes of follow-up. Because ACI has high recurrence rate and mortality, it is necessary to carry out follow-up to investigate patients' conditions after treatment. In consideration of outcomes, we suggested that clinical trials should report long-term end-points and valuable outcomes. Thirdly, there were selection bias and performance bias among included RCTs in current NMA, as several RCTs did not use the blinding or was not registered in advance. Besides, the consistency test was also showed that there was certain heterogeneity between direct and indirect comparisons. These elements may cause non-standardization of RCTs and generate an overestimate for eligible CHIs. In terms of above limitations, the RCTs that will be conducted in the future should be refined in relevant areas.

## 5. Conclusion

In summary, this NMA found that STS plus WM was the optimum treatment regimen for patients with ACI. Also DH and DSSA were better choices for treating ACI as well. On account of the limitations of included RCTs, more high quality and multicenter RCTs are needed to support the findings of this NMA in the future.

## Figures and Tables

**Table 1:** Results of the network meta-analysis of clinical effectiveness rate (lower left quarter) and neurological impairment (upper right quarter).

**A**	-0.37 (-2.82, 2.04)	-1.16 (-2.39, 0.07)	-4.38 (-5.07, -3.70)	-4.52 (-5.65, -3.4)	-5.01 (-7.19, -2.84)	-3.45 (-4.96, -1.96)	-2.56 (-5.40, 0.30)	-

1.64 (1.09, 2.55)	**B**	-0.79 (-3.41, 1.84)	-4.01 (-6.40, -1.60)	-4.15 (-6.70, -1.60)	-4.64 (-7.89, -1.38)	-3.08 (-5.94, -0.22)	-2.19 (-5.92, 1.56)	-

1.40 (1.11, 1.74)	0.89 (0.54, 1.35)	**C**	-3.22 (-4.40, -2.04)	-3.37 (-4.78, -1.93)	-3.86 (-6.4, -1.36)	-2.29 (-4.22, -0.35)	-1.41 (-4.51, 1.70)	-

4.03 (3.41, 4.69)	2.56 (1.65, 3.70)	2.90 (2.31, 3.59)	**D**	-0.14 (-1.38, 1.11)	-0.63 (-2.91, 1.67)	0.93 (-0.70, 2.57)	1.82 (-1.11, 4.74)	-

3.61 (2.80, 4.61)	2.30 (1.40, 3.46)	2.60 (1.95, 3.48)	0.90 (0.69, 1.17)	**E**	-0.49 (-2.96, 1.97)	1.07 (-0.80, 2.96)	1.96 (-1.10, 5.01)	-

4.15 (2.84, 5.87)	2.65 (1.44, 4.31)	3.00 (1.93, 4.43)	1.04 (0.68, 1.53)	1.17 (0.73, 1.81)	**F**	1.56 (-1.07, 4.21)	2.45 (-1.15, 6.04)	-

3.15 (2.35, 4.23)	2.02 (1.12, 3.19)	2.28 (1.55, 3.29)	0.79 (0.56, 1.10)	0.89 (0.58, 1.29)	0.79 (0.48, 1.22)	**G**	0.89 (-2.31, 4.13)	-

5.52 (1.37, 17.00)	3.54 (0.80, 11.3)	4.00 (0.96, 12.74)	1.38 (0.31, 4.46)	1.56 (0.37, 4.82)	1.38 (0.31, 4.46)	1.79 (0.42, 5.61)	**H**	-

922.30 (2.05, 424.80)	672.30 (1.27, 271.20)	668.30 (1.52, 296.00)	228.10 (0.52, 107.60)	272.00 (0.57, 119.70)	234.10 (0.49, 103.90)	312.20 (0.64, 132.8)	300.80 (0.32, 128.70)	**I**

A: WM; B: Danshen injection + WM; C: Fufang Danshen injection + WM; D: Danhong injection + WM; E: Danshenchuanxiongqin injection + WM; F: Sodium Tanshinone IIA Sulfonate injection + WM; G: Salvianolate injection + WM; H: Danshen Salvianolic Acids injection + WM; I: Guanxinning injection + WM. The result underlined meant it had statistical significant.

**Table 2:** Results of the network meta-analysis of activities of daily living function (lower left quarter) and whole blood high shear viscosity (upper right quarter).

**A**	-1.32 (-2.37, -0.27)	-0.42 (-1.48, 0.64)	-1.55 (-2.37, -0.74)	-1.50 (-3.82, 0.81)	-1.66 (-3.11, -0.21)	-0.72 (-1.75, 0.29)	-0.57 (-2.65, 1.5)

1.63 (-5.61, 8.88)	**B**	0.90 (-0.50, 2.31)	-0.23 (-1.43, 0.98)	-0.17 (-2.69, 2.30)	-0.34 (-2.13, 1.43)	0.60 (-0.86, 2.06)	0.75 (-1.59, 3.09)

10.83 (4.68, 16.87)	9.20 (-0.07, 18.32)	**C**	-1.13 (-1.95, -0.33)	-1.08 (-3.15, 0.96)	-1.24 (-3.02, 0.59)	-0.30 (-1.76, 1.15)	-0.15 (-2.5, 2.2)

10.55 (7.42, 13.63)	8.92 (1.74, 16.10)	-0.28 (-6.79, 6.30)	**D**	0.05 (-2.17, 2.24)	-0.11 (-1.75, 1.54)	0.83 (-0.46, 2.12)	0.98 (-1.25, 3.23)

9.53 (5.48, 13.60)	7.90 (0.82, 14.96)	-1.30 (-8.08, 5.61)	-1.02 (-5.92, 3.99)	**E**	-0.16 (-2.85, 2.56)	0.77 (-1.73, 3.32)	0.92 (-2.21, 4.05)

16.53 (6.94, 26.17)	14.91 (2.97, 26.98)	5.71 (-5.55, 17.14)	5.99 (-4.17, 16.14)	7.01 (-3.34, 17.37)	**F**	0.94 (-0.82, 2.71)	1.09 (-1.42, 3.61)

7.37 (2.35, 12.45)	5.74 (-3.14, 14.48)	-3.46 (-11.26, 4.55)	-3.18 (-9.11, 2.82)	-2.16 (-8.66, 4.27)	-9.17 (-19.95, 1.71)	**G**	0.15 (-2.15, 2.44)

9.73 (4.00, 15.40)	8.11 (-1.05, 17.32)	-1.10 (-9.46, 7.35)	-0.82 (-7.37, 5.76)	0.21 (-6.83, 7.23)	-6.80 (-18.03, 4.38)	2.37 (-5.32, 10.08)	**H**

A: WM; B: Danshen injection + WM; C: Fufang Danshen injection + WM; D: Danhong injection + WM; E: Danshenchuanxiongqin injection + WM; F: Sodium Tanshinone IIA Sulfonate injection + WM; G: Salvianolate injection + WM; H: Danshen Salvianolic Acids injection + WM. The result underlined meant it had statistical significant.

**Table 3:** Results of the network meta-analysis of the whole blood low shear viscosity (lower left quarter) and plasma viscosity (upper right quarter).

**A**	-0.34 (-0.67, -0.02)	-0.01 (-0.38, 0.36)	-0.53 (-0.79, -0.25)	-0.38 (-0.95, 0.2)	-	-0.15 (-0.54, 0.23)	-

-1.72 (-6.44, 2.96)	**B**	0.33 (-0.1, 0.77)	-0.19 (-0.52, 0.15)	-0.04 (-0.61, 0.54)	-	0.19 (-0.31, 0.69)	-

-0.09 (-4.73, 4.53)	1.63 (-4.52, 7.82)	**C**	-0.52 (-0.81, -0.23)	-0.37 (-1.03, 0.29)	-	-0.14 (-0.68, 0.39)	-

-1.33 (-4.91, 2.24)	0.39 (-4.94, 5.74)	-1.24 (-4.71, 2.27)	**D**	0.15 (-0.46, 0.75)	-	0.37 (-0.1, 0.85)	-

-1.26 (-11.51, 9.03)	0.46 (-10.67, 11.54)	-1.16 (-10.34, 7.99)	0.08 (-9.83, 9.94)	**E**	-	0.22 (-0.46, 0.91)	-

-19.89 (-26.42, -13.4)	-18.17 (-26.23, -10.16)	-19.79 (-27.82, -11.77)	-18.55 (-25.93, -11.24)	-18.63 (-30.79, -6.51)	**F**	-	-

-1.33 (-5.99, 3.3)	0.39 (-6.31, 6.93)	-1.24 (-7.72, 5.4)	0 (-5.83, 5.86)	-0.08 (-11.28, 11.1)	18.55 (10.54, 26.64)	**G**	-

-0.59 (-9.67, 8.64)	1.13 (-9.13, 11.34)	-0.49 (-10.73, 9.79)	0.75 (-9.08, 10.55)	0.67 (-13.1, 14.53)	19.3 (8.18, 30.54)	0.75 (-9.4, 11.15)	**H**

A: WM; B: Danshen injection + WM; C: Fufang Danshen injection + WM; D: Danhong injection + WM; E: Danshenchuanxiongqin injection + WM; F: Sodium Tanshinone IIA Sulfonate injection + WM; G: Salvianolate injection + WM; H: Danshen Salvianolic Acids injection + WM. The result underlined meant it had statistical significant.

**Table 4:** Results of the network meta-analysis of the fibrinogen (lower left quarter).

**A**							

-0.25 (-0.91, 0.40)	**B**						

0.01 (-0.40, 0.44)	0.27 (-0.39, 0.93)	**C**					

-1.09 (-1.37, -0.80)	-0.83 (-1.45, -0.22)	-1.10 (-1.43, -0.78)	**D**				

-0.36 (-0.92, 0.21)	-0.10 (-0.74, 0.53)	-0.37 (-0.94, 0.21)	0.73 (0.18, 1.29)	**E**			

-1.13 (-1.95, -0.29)	-0.88 (-1.92, 0.18)	-1.14 (-2.06, -0.22)	-0.04 (-0.91, 0.83)	-0.77 (-1.77, 0.24)	**F**		

-0.44 (-0.98, 0.10)	-0.19 (-1.05, 0.66)	-0.45 (-1.14, 0.23)	0.65 (0.03, 1.26)	-0.08 (-0.87, 0.69)	0.69 (-0.31, 1.65)	**G**	

-1.79 (-3.51, -0.06)	-1.54 (-3.39, 0.32)	-1.8 (-3.58, -0.02)	-0.70 (-2.45, 1.06)	-1.43 (-3.25, 0.39)	-0.66 (-2.58, 1.27)	-1.35 (-3.14, 0.47)	**H**

A: WM; B: Danshen injection + WM; C: Fufang Danshen injection + WM; D: Danhong injection + WM; E: Danshenchuanxiongqin injection + WM; F: Sodium Tanshinone IIA Sulfonate injection + WM; G: Salvianolate injection + WM; H: Danshen Salvianolic Acids injection + WM. The result underlined meant it had statistical significant.

## Abbreviations

95% CIs: 95% confidence intervals
ACI: Acute cerebral infarction
ADRs/ADEs: Adverse drug reactions/adverse drug events
CHIs: Chinese herbal injections
DH: Danhong injection
DS: Danshen injection
DSCIs: Danshen class injections
DSCXQ: Danshenchuanxiongqin injection
DSSA: Danshen Salvianolic Acids injection
FFDS: Fufang Danshen injection
GXN: Guanxinning injection
IF: Inconsistency factor
NMA: Network meta-analysis
MD: Mean difference
OR: Odds ratio
RCTs: Randomized controlled trials
SI: Salvianolate injection
STS: Sodium Tanshinone IIA Sulfonate injection
SUCRA: Surface under the cumulative ranking probabilities
WM: Western medicine.

## Supplementary Materials

Supplementary Materials Table S1. PRISMA checklist for network meta-analysis. Table S2. Search strategy. Table S3. The basic characteristics of the included studies.
