# Supplementary material for: Efficacy of Danshen Class Injection in the Treatment of Acute Cerebral Infarction: A Bayesian Network Meta-Analysis of Randomized Controlled Trials
Source: Evid Based Complement Alternat Med. 2019 Feb 3;2019:5814749. doi: 10.1155/2019/5814749 (PMC6377994; doi:10.1155/2019/5814749)
Supplement: Supplementary Materials — Table S1. PRISMA checklist for network meta-analysis. Table S2. Search strategy. Table S3. The basic characteristics of the included studies. [file 5814749.f1.doc]

**Supplementary Table S1: PRISMA Checklist for Network Meta-analysis**

| **Section/topic** | **#** | **Checklist item** | **Reported on page #** |
| --- | --- | --- | --- |
| **TITLE** | | |  |
| Title | 1 | Identify the report as a systematic review incorporating a network meta-analysis (or related form of  meta-analysis). | 1 |
| **ABSTRACT** | | |  |
| Structured summary | 2 | Provide a structured summary including, as applicable:  Background: main objectives  Methods: data sources; study eligibility criteria, participants, and interventions; study appraisal; and synthesis methods, such as network meta-analysis.  Results: number of studies and participants identified; summary estimates with corresponding confidence/credible intervals; treatment rankings may also be discussed. Authors may choose to summarize pairwise comparisons against a chosen treatment included in their analyses for brevity.  Discussion/Conclusions: limitations; conclusions and implications of findings.  Other: primary source of funding; systematic review registration number with registry name. | 1 |
| **INTRODUCTION** | | |  |
| Rationale | 3 | Describe the rationale for the review in the context of what is already known, including mention of why a network meta-analysis has been conducted | 1-2 |
| Objectives | 4 | Provide an explicit statement of questions being addressed with reference to participants, interventions, comparisons, outcomes, and study design (PICOS). | 1-2 |
| **METHODS** | | |  |
| Protocol and registration | 5 | Indicate if a review protocol exists and if and where it can be accessed (e.g., Web address), and, if available, provide registration information including registration number. | / |
| Eligibility criteria | 6 | Specify study characteristics (e.g., PICOS, length of follow-up) and report characteristics (e.g., years considered, language, publication status) used as criteria for eligibility, giving rationale. Clearly describe eligible treatments included in the treatment network, and note whether any have been clustered or merged into the same node (with justification). | 3-4 |
| Information sources | 7 | Describe all information sources (e.g., databases with dates of coverage, contact with study authors to identify additional studies) in the search and date last searched. | 4 |
| Search | 8 | Present full electronic search strategy for at least one database, including any limits used, such that it could be repeated. | 4-5 |
| Study selection | 9 | State the process for selecting studies (i.e., screening, eligibility, included in systematic review, and, if applicable, included in the meta-analysis). | 4-5 |
| Data collection process | 10 | Describe method of data extraction from reports (e.g., piloted forms, independently, in duplicate) and any processes for obtaining and confirming data from investigators. | 4 |
| Data items | 11 | List and define all variables for which data were sought (e.g., PICOS, funding sources) and any assumptions and simplifications made. | 4 |
| Geometry of the network | 12 | Describe methods used to explore the geometry of the treatment network under study and potential biases related to it. This should include how the evidence base has been graphically summarized for presentation, and what characteristics were compiled and used to describe the evidence base to readers | 5 |
| Risk of bias within individual studies | 13 | Describe methods used for assessing risk of bias of individual studies (including specification of whether this was done at the study or outcome level), and how this information is to be used in any data synthesis. | 5 |
| Summary measures | 14 | State the principal summary measures (e.g., risk ratio, difference in means). Also describe the use of additional summary measures assessed, such as treatment rankings and surface under the cumulative ranking curve (SUCRA) values, as well as modified approaches used to present summary findings from meta-analyses. | 5 |
| Planned methods of analysis | 15 | Describe the methods of handling data and combining results of studies for each network meta-analysis. This should include, but not be limited to: Handling of multigroup trials; Selection of variance structure; Selection of prior distributions in Bayesian analyses; and Assessment of model fit. | 5 |
| Assessment of inconsistency | 16 | Describe the statistical methods used to evaluate the agreement of direct and indirect evidence in the treatment network(s) studied. Describe efforts taken to address its presence when found. | 5 |
| Risk of bias across studies | 17 | Specify any assessment of risk of bias that may affect the cumulative evidence (e.g., publication bias, selective reporting within studies) | 5 |
| Additional analyses | 18 | Describe methods of additional analyses if done, indicating which were prespecified. This may include, but not be limited to, the following: Sensitivity or subgroup analyses; Meta-regression analyses; Alternative formulations of the treatment network; and Use of alternative prior distributions for Bayesian analyses (if applicable). | 5 |
| **RESULTS** | | |  |
| Study selection | 19 | Give numbers of studies screened, assessed for eligibility, and included in the review, with reasons for exclusions at each stage, ideally with a flow diagram. | 6 |
| Presentation of network structure | 20 | Provide a network graph of the included studies to enable visualization of the geometry of the treatment network. | 6 |
| Summary of network geometry | 21 | Provide a brief overview of characteristics of the treatment network. This may include commentary on the abundance of trials and randomized patients for the different interventions and pairwise comparisons in the network, gaps of evidence in the treatment network, and potential biases reflected by the network structure. | 6 |
| Study characteristics | 22 | For each study, present characteristics for which data were extracted (e.g., study size, PICOS, follow-up period) and provide the citations. | 6 |
| Risk of bias within studies | 23 | Present data on risk of bias of each study and, if available, any outcome level assessment. | 6 |
| Results of individual studies | 24 | For all outcomes considered (benefits or harms), present, for each study: 1) simple summary data for each intervention group, and 2) effect estimates and confidence intervals. Modified approaches may be needed to deal with information from larger networks. | 6-10 |
| Synthesis of results | 25 | Present results of each meta-analysis done, including confidence/credible intervals. In larger networks, authors may focus on comparisons versus a particular comparator (e.g., placebo or standard care), with full findings presented in an appendix. League tables and forest plots may be considered to summarize pairwise comparisons. If additional summary measures were explored (such as treatment rankings), these should also be presented. | 6-10 |
| Exploration for inconsistency | 26 | Describe results from investigations of inconsistency. This may include such information as measures of model fit to compare consistency and inconsistency models, P values from statistical tests, or summary of inconsistency estimates from different parts of the treatment network. | 10 |
| Risk of bias across studies | 27 | Present results of any assessment of risk of bias across studies for the evidence base being studied. | 6 |
| Results of additional analyses | 28 | Give results of additional analyses, if done (e.g., sensitivity or subgroup analyses, meta-regression  analyses, alternative network geometries studied, alternative choice of prior distributions for  Bayesian analyses, and so forth). | 10 |
| **DISCUSSION** | | |  |
| Summary of evidence | 29 | Summarize the main findings, including the strength of evidence for each main outcome; consider their relevance to key groups (e.g., health care providers, researchers, and policymakers). | 12 |
| Limitations | 30 | Discuss limitations at study and outcome level (e.g., risk of bias), and at review level (e.g., incomplete retrieval of identified research, reporting bias). Comment on the validity of the assumptions, such as transitivity and consistency. Comment on any concerns regarding network geometry (e.g., avoidance of certain comparisons). | 12 |
| Conclusions | 31 | Provide a general interpretation of the results in the context of other evidence, and implications for future research. | 12 |
| **FUNDING** | | |  |
| Funding | 32 | Describe sources of funding for the systematic review and other support (e.g., supply of data); role of funders for the systematic review. This should also include information regarding whether funding has been received from manufacturers of treatments in the network and/or whether some of the authors are content experts with professional conflicts of interest that could affect use of treatments in the network. | 1 |

| **Supplementary Table S2: Search Strategy** |
| --- |
| #1 "Brain Infarction"[MeSH Terms]  #2 "cerebral infarction"[Title/Abstract] OR "stoke"[Title/Abstract] OR "brain embolism"[Title/Abstract] OR "Ischemic stroke"[Title/Abstract] OR "cerebrovascular disorders"[Title/Abstract]  #3 #1 OR #2  #4 salvia militorrhizo injection"[Title/Abstract] OR "danshen injection"[Title/Abstract]) OR "danshen zhusheye"[Title/Abstract]  #5 "compound danshen injection"[Title/Abstract] OR "composite salvia miltiorrhiza injection"[Title/Abstract] OR "fufang danshen injection"[Title/Abstract] OR "fufang danshen  zhusheye"[Title/Abstract]  #6 "danhong injection "[Title/Abstract] OR "danhong zhusheye"[Title/Abstract]  #7 "danshenchuanxiongqin"[Title/Abstract] OR "salvia ligustrazin injection"[Title/Abstract]  #8 "salvianolate injection"[Title/Abstract] OR "zhu she yong danshenduofensuan"[Title/Abstract]  #9 "danshen salvianolic acids injection "[Title/Abstract] OR "danshenduofensuanyan zhusheye"[Title/Abstract]  #10 "Tanshinone Ⅱ A injection"[Title/Abstract]  #11 "Guanxinning injection"[Title/Abstract]  #12 #4 OR #5 OR #6 OR #7 OR #8 OR #9 OR #10 OR #11  #13 randomized controlled trial [Publication Type]  #14 controlled clinical trial [Publication Type]  #15 randomized [Title/Abstract]  #16 placebo [Title/Abstract]  #17 randomly [Title/Abstract]  #18 trial [Title/Abstract]  #19 groups[Title/Abstract]  #20 drug therapy [MeSH Terms]  #21 "drug therapy" [Subheading]  #22 #13 OR #14 OR #15 OR #16 OR #17 OR #18 OR #19 OR #20 OR #21  #23 animals [MeSH Terms]  #24 humans [MeSH Terms]  #25 #23 NOT #24  #26 #22 NOT #25  #27 #3 AND #12 AND #26 |

| **Supplementary Table S3. Characteristics of included studies** | | | | | | | | | | | | |
| --- | --- | --- | --- | --- | --- | --- | --- | --- | --- | --- | --- | --- |
| Study-ID | Disease time | | N(E/C） | | Sex  (M/F) | Age | Therapy of experiment | Therapy of control | | Course | Outcomes | Jadad score |
| Li 2007 | | 72h | | 32/30 | 35/27 | 56.3 40-75 | DS400mg+WM | | WM | 14d | ①④⑤⑥ | 2 |
| Shen 2007 | | 48h | | 30/30 | 37/23 | 45-80 | DS16ml+WM | | WM | 14d | ②④⑤⑥⑧ | 1 |
| Xia 2016 | | 72h | | 60/60 | 72/48 | 63.1 41-78 | DS30ml+WM | | WM | 14d | ①④⑤⑥⑧ | 4 |
| Yu 2010 | | 48h | | 35/34 | 44/25 | 62.1 | DS20ml+WM | | WM | 14d | ① | 4 |
| Hao 2005 | | 24h | | 43/41 | 56/28 | 54.3 | FFDS20ml+WM | | WM | 14d | ①④⑤⑥ | 2 |
| Li 2014 | | 8-72h | | 36/36 | 46/26 | 58.4 52-74 | FFDS20ml+WM | | WM | 14d | ①②⑧ | 4 |
| Liang 2016 | | 72h | | 65/65 | 77/53 | 57.4 45-75 | FFDS20ml+WM | | WM | 14d | ①②③ | 4 |
| Lv 2014 | | 72h | | 84/84 | 100/68 | 57.4 | FFDS10-20mlWM | | WM | 14d | ① | 4 |
| Shi 2012 | | 72h | | 32/32 | 36/28 | 70.5 65-81 | FFDS16ml+WM | | WM | 14d | ①②③ | 4 |
| Zhang 2007 | | 72h | | 43/43 | 57/29 | 56.3 45-79 | FFDS40ml+WM | | WM | 14d | ①②⑧ | 4 |
| Zheng 2014 | | 72h | | 38/38 | 37/39 | 62.1 35-76 | FFDS10ml+WM | | WM | 14d | ①② | 2 |
| An 2015 | | 48h | | 35/35 | 42/28 | 58.5 43-80 | DH30ml+WM | | WM | 14d | ①②⑧ | 2 |
| Chen 2013 | | 72h | | 70/64 | 67/67 | 44-79 | DH40ml+WM | | WM | 14d | ①②⑧ | 2 |
| Cheng 2010 | | 24h | | 43/40 | 49/34 | 63.1 | DH20ml+WM | | WM | 14d | ① | 2 |
| Cui 2014 | | 72h | | 40/40 | 47/33 | 62.7 33-80 | DH20ml+WM | | WM | 15d | ①②③ | 2 |
| Du 2011 | | 72h | | 57/55 | 73/39 | 66.8 46-79 | DH50-100ml+WM | | WM | 14d | ②⑦ | 2 |
| Gu 2012 | | 72h | | 80/80 | 79/81 | 64.6 | DH30ml+WM | | WM | 15d | ①②④⑤⑥⑦⑧ | 2 |
| Gu 2014 | | 24h | | 65/60 | 77/48 | 70.7 62-80 | DH20ml+WM | | WM | 14d | ①②④⑤⑥⑦ | 4 |
| Han 2009 | | 72h | | 30/30 | 34/26 | 68.5 52-78 | DH40ml+WM | | WM | 14d | ② | 2 |
| Hao 2008 | | 48h | | 124/120 | 167/77 | 60.0 37-81 | DH100ml+WM | | WM | 14d | ①⑧ | 3 |
| Hao 2013 | | 72h | | 62/62 | 68/56 | 68 | DH40ml+WM | | WM | 14d | ①②⑧ | 2 |
| Hu 2008 | | 72h | | 32/32 | 35/29 | 65.5 56-75 | DH40ml+WM | | WM | 14d | ②④⑤⑥⑧ | 2 |
| Jiang 2008 | | 72h | | 40/40 | 47/33 | 59.6 35-85 | DH30ml+WM | | WM | 14d | ①② | 2 |
| Jiang 2011 | | 48h | | 31/28 | 28/31 | 50-79 | DH40ml+WM | | WM | 14d | ① | 2 |
| Li 2010 | | 72h | | 30/30 | 39/21 | 63.0 39-81 | DH30ml+WM | | WM | 14d | ①②③ | 2 |
| Li 2015 | | 72h | | 40/40 | 47/33 | 62.9 48-79 | DH40ml+WM | | WM | 14d | ② | 2 |
| Li 2012 | | 72h | | 36/32 | 37/31 | 64.3 51-78 | DH20ml+WM | | WM | 14d | ①②⑦⑧ | 2 |
| Li 2012 | | 72h | | 40/30 | 45/25 | 47-70 | DH40-60ml+WM | | WM | 28d | ①② | 2 |
| Li 2011 | | 48h | | 32/32 | 41/23 | 66.5 42-79 | DH20ml+WM | | WM | 14d | ①②⑧ | 2 |
| Li 2015 | | 72h | | 60/60 | 70/50 | 65.7 50-78 | DH40ml+WM | | WM | 14d | ① | 2 |
| Li 2014 | | 24h | | 40/40 | 63/17 | 48-76 | DH30ml+WM | | WM | 14d | ② | 2 |
| Li 2010 | | 2w | | 33/33 | 39/27 | 63.5 43-78 | DH30ml+WM | | WM | 21d | ①⑦⑧ | 2 |
| Liang 2014 | | 26h | | 45/45 | 41/49 | 55.5 40-67 | DH30ml+WM | | WM | 14d | ①② | 4 |
| Liu 2013 | | 6h-1w | | 59/59 | - | 59.1 40-76 | DH20ml+WM | | WM | 14d | ①②⑦ | 2 |
| Liu 2008 | | 72h | | 56/54 | 57/53 | 71.1 48-79 | DH30ml+WM | | WM | 14d | ②③ | 2 |
| Liu 2010 | | 72h | | 40/40 | 45/35 | 51-71 | DH40ml+WM | | WM | 15d | ①⑧ | 2 |
| Luo 2012 | | 72h | | 90/90 | - | - | DH40ml+WM | | WM | 14d | ①②⑦ | 4 |
| Ma 2015 | | 72h | | 45/45 | 49/41 | 65.5 45-79 | DH20ml+WM | | WM | 28d | ①③④⑤⑥ | 4 |
| Mao 2010 | | 72h | | 29/29 | 35/23 | 61.2 43-75 | DH20ml+WM | | WM | 14d | ① | 4 |
| Mu 2015 | | 72h | | 30/30 | 31/29 | 69.13 | DH20ml+WM | | WM | 14d | ①②⑧ | 2 |
| Ni 2015 | | 7h-7d | | 60/60 | 80/40 | 43.8 46-79 | DH30ml+WM | | WM | 14d | ①②⑧ | 2 |
| Qi 2013 | | 48h | | 40/40 | 47/33 | 56 42-79 | DH20ml+WM | | WM | 14d | ① | 2 |
| Qiao 2010 | | 7d | | 30/30 | 39/21 | 43-80 | DH20ml+WM | | WM | 14d | ①② | 2 |
| Shen 2012 | | 48h | | 36/36 | 34/38 | 60.4 39-78 | DH30ml+WM | | WM | 14d | ②③⑧ | 4 |
| Li 2012 | | 7-96 | | 38/37 | 43/32 | 62.2 42-85 | DH40ml+WM | | WM | 14d | ①②④⑤⑥⑦⑧ | 3 |
| Tang 2008 | | 72h | | 40/40 | 48/32 | 65.3 | DH40ml+WM | | WM | 14d | ②⑧ | 2 |
| Tao 2011 | | 48h | | 55/20 | 46/29 | 55.9 42-78 | DH30ml+WM | | WM | 15d | ①②⑥⑦ | 2 |
| Tao 2012 | | 6-96h | | 30/30 | 37/23 | 40-79 | DH20ml+WM | | WM | 14d | ①②⑦ | 2 |
| Tian 2010 | | 48h | | 36/34 | 39/31 | 60.5 47-70 | DH30ml+WM | | WM | 15d | ①②⑧ | 2 |
| Wang 2015 | | 72h | | 50/50 | 54/46 | 62.5 36-85 | DH20ml+WM | | WM | 28d | ①②③ | 2 |
| Wang 2008 | | 72h | | 30/30 | 32/28 | 61.3 | DH40ml+WM | | WM | 14d | ② | 2 |
| Wang 2011 | | 24h | | 40/40 | 46/34 | 61.2 42-78 | DH20ml+WM | | WM | 21d | ②③ | 2 |
| Wang 2015 | | 48h | | 64/64 | 94/34 | 61 43-73 | DH40ml+WM | | WM | 14d | ②⑧ | 2 |
| Wang 2008 | | 7d | | 50/50 | 48/52 | 50-79 | DH20ml+WM | | WM | 14d | ①② | 2 |
| Wu 2012 | | 48h | | 48/46 | 55/39 | 65.0 52-81 | DH20ml+WM | | WM | 14d | ①② | 2 |
| Wu 2013 | | 72h | | 30/30 | 35/25 | 71.2 50-80 | DH30ml+WM | | WM | 14d | ① | 2 |
| Wu 2014 | | 72h | | 42/42 | 46/38 | 57 38-72 | DH30ml+WM | | WM | 14d | ①②⑧ | 2 |
| Xie 2008 | | 7d | | 30/30 | 39/21 | 44-79 | DH20ml+WM | | WM | 14d | ①② | 2 |
| Xu 2014 | | 72h | | 45/45 | 70/20 | 71.1 56-79 | DH20ml+WM | | WM | 14d | ①②⑧ | 2 |
| Xuan 2011 | | 48h | | 60/60 | 63/57 | 44-73 | DH20ml+WM | | WM | 14d | ① | 2 |
| Yan 2013 | | 72h | | 57/59 | 63/53 | 41-73 | DH30ml+WM | | WM | 14d | ①②⑧ | 2 |
| Yang 2015 | | 72h | | 49/49 | 56/42 | 62.2 54-78 | DH20ml+WM | | WM | 14d | ①②③ | 2 |
| Yin 2010 | | 24h | | 40/40 | 43/37 | 62.7 40-76 | DH30ml+WM | | WM | 14d | ①② | 2 |
| Zhang 2012 | | 72h | | 64/57 | 81/40 | 70.5 51-79 | DH30ml+WM | | WM | 14d | ①② | 2 |
| Zhang 2008 | | 48h | | 40/40 | 47/33 | 66.0 56-76 | DH20ml+WM | | WM | 14d | ①②⑧ | 3 |
| Zhang 2015 | | 72h | | 40/40 | 44/36 | 60.7 40-75 | DH20ml+WM | | WM | 14d | ②③ | 3 |
| Zhang 2011 | | 72h | | 50/48 | 61/37 | 55.5 44-77 | DH20ml+WM | | WM | 14d | ①② | 2 |
| Zhang 2013 | | 72h | | 40/40 | 46/34 | 66.8 | DH20ml+WM | | WM | 14d | ① | 4 |
| Zhang 2015 | | 24h | | 90/90 | 88/92 | 51.2 45-67 | DH40mg+WM | | WM | 14d | ①② | 2 |
| Zhang 2011 | | 72h | | 59/38 | 53/44 | 61-78 | DH20ml+WM | | WM | 14d | ①②③ | 2 |
| Zhang 2010 | | 48h | | 60/60 | 78/42 | 69.1 42-80 | DH40ml+WM | | WM | 14d | ① | 2 |
| Zhong 2011 | | 72h | | 35/35 | 33/37 | 72.0 61-78 | DH20ml+WM | | WM | 14d | ①②⑧ | 2 |
| Zhou 2007 | | 7d | | 30/30 | 39/21 | 43-80 | DH20ml+WM | | WM | 14d | ①② | 2 |
| Fang 2013 | | 72h | | 48/48 | 50/46 | 50.6 52-64 | DH20ml+WM | | WM | 14d | ①② | 4 |
| Li 2013 | | 12h | | 68/50 | 63/55 | 64.0 56-69 | DH40ml+WM | | WM | 14d | ①② | 2 |
| Zhu 2012 | | 72h | | 46/46 | - | <80 | DH40ml+WM | | WM | 14d | ②③⑧ | 2 |
| Guo 2015 | | 48h | | 48/48 | 51/45 | 63.2 | DSCXQl0ml+WM | | WM | 14d | ①②⑧ | 2 |
| Lan 2015 | | 72h | | 40/40 | 41/39 | 63.0 54-72 | DSCXQ10ml+WM | | WM | 14d | ①②③⑧ | 2 |
| Li 2014 | | 7d | | 60/60 | 72/48 | 64.7 45-79 | DSCXQ10ml+WM | | WM | 14d | ① | 3 |
| Ling 2013 | | 7d | | 40/40 | 47/33 | 57.3 | DSCXQ10ml+WM | | WM | 14d | ① | 2 |
| Liu 2011 | | 72h | | 34/33 | 39/28 | 60.5 40-80 | DSCXQ10ml+WM | | WM | 14d | ① | 2 |
| Liu 2015 | | 72h | | 30/30 | 32/28 | 59.2 48-72 | DSCXQ10ml+WM | | WM | 10d | ① | 2 |
| Liu 2014 | | 48h | | 68/68 | 79/57 | 60.1 40-72 | DSCXQ10ml+WM | | WM | 14d | ①②⑧ | 2 |
| Wan 2015 | | 12h | | 41/41 | 48/34 | 60.3 48-76 | DSCXQ10ml+WM | | WM | 28d | ①③ | 4 |
| Wang 2015 | | 48h | | 64/64 | 71/57 | 60.6 46-75 | DSCXQ10ml+WM | | WM | 14d | ①②⑥⑦⑧ | 4 |
| Wang 2012 | | 48h | | 40/40 | 46/34 | 64.2 52-73 | DSCXQ20ml+WM | | WM | 14d | ①②⑧ | 2 |
| Yang 2010 | | 72h | | 31/31 | 41/21 | 57.5 50-65 | DSCXQ10ml+WM | | WM | 14d | ① | 2 |
| Ye 2013 | | 72h | | 40/40 | 42/38 | 65.0 | DSCXQl0ml+WM | | WM | 14d | ①② | 2 |
| Zhang 2012 | | 48h | | 40/40 | 52/28 | 57.5 | DSCXQ10ml+WM | | WM | 14d | ①②⑧ | 3 |
| Zhao 2015 | | 7d | | 60/60 | 71/49 | 56.8 38-78 | DSCXQ10ml+WM | | WM | 28d | ①②③ | 2 |
| Zhao 2013 | | 48h | | 55/55 | 63/47 | 63.7 55-72 | DSCXQ10ml+WM | | WM | 14d | ①②⑧ | 3 |
| Hu 2015 | | 72h | | 40/40 | 42/38 | 65.0 | DSCXQ10ml+WM | | WM | 14d | ①② | 4 |
| Wang 2016 | | 72h | | 56/56 | 60/52 | 60.8 49-73 | DSCXQ10ml+WM | | WM | 14d | ①②③⑧ | 2 |
| Li 2016 | | 48h | | 84/84 | 106/62 | 67.7 61-79 | DSCXQ10ml+WM | | WM | 14d | ①②③ | 4 |
| Gong 2009 | | 48 | | 32/32 | 35/29 | 62 49-80 | STS60 mg+WM | | WM | 14d | ①②⑧ | 2 |
| Gong 2010 | | 48 | | 45/60 | 68/37 | 64.9 | STS40mg+WM | | WM | 14d | ①② | 2 |
| Kang 2015 | | 12 | | 50/50 | 73/27 | 63.2 | STS80mg+WM | | WM | 14d | ①⑧ | 4 |
| Li 2016 | | 24 | | 123/123 | 139/107 | 63.74 | STS15mg+WM | | WM | 21d | ①④⑤⑦ | 4 |
| Ma 2012 | | 48 | | 85/85 | 89/81 | 49-78 | STS40mg+WM | | WM | 14d | ①②⑧ | 2 |
| Tan 2008 | | 72 | | 42/40 | 57/25 | 59.9 | STS40mg+WM | | WM | 14d | ①②⑧ | 2 |
| Xu 2015 | | 48 | | 53/53 | 69/37 | 64.78 | STS60mg+WM | | WM | 14d | ①② | 4 |
| Yang 2013 | | 24 | | 30/30 | 34/26 | 48-85 | STS6 mg+WM | | WM | 10d | ①⑧ | 2 |
| Zhang 2016 | | 72 | | 36/36 | 37/35 | 64.3 48-72 | STS40mg+WM | | WM | 14d | ①④⑤ | 2 |
| Zhang 2008 | | 72 | | 50/54 | 64/40 | 63.1 | STS8ml+WM | | WM | 14d | ①②③⑧ | 2 |
| Zhou 2014 | | 72 | | 20/20 | 24/16 | 65.5 55-75 | STS40mg+WM | | WM | 14d | ①⑧ | 2 |
| Dan 2016 | | 72h | | 74/74 | 81/67 | 61.1 46-78 | SI0.2g+WM | | WM | 21d | ② | 2 |
| Duan 2013 | | 72h | | 42/42 | 61/23 | 64.2 45-80 | SI0.2g+WM | | WM | 14d | ② | 2 |
| Hao 2012 | | 72h | | 30/30 | 31/29 | 61.1 44-72 | SI0.2g+WM | | WM | 14d | ①② | 2 |
| Ji 2010 | | 72h | | 93/52 | 74/74 | 60.9 40-75 | SI0.3g+WM | | WM | 15d | ②③⑧ | 5 |
| Li 2016 | | 72h | | 42/41 | 55/28 | 62.1 44-82 | SI0.2g+WM | | WM | 14d | ①② | 4 |
| Mi 2012 | | 72h | | 150/150 | 172/128 | 63.8 | SI0.2g+WM | | WM | 14d | ①④⑤ | 2 |
| Nie 2015 | | 16h | | 89/89 | 102/76 | 65.4 55-85 | SI0.2g+WM | | WM | 15d | ①②⑧ | 2 |
| Yu 2014 | | 24h | | 80/80 | 81/79 | 66.6 50-80 | SI0.2g+WM | | WM | 14d | ①②③⑧ | 2 |
| Tao 2015 | | 72h | | 49/49 | 56/42 | 66.8 60-78 | SI0.2g+WM | | WM | 14d | ②③④⑤⑥ | 2 |
| Wang 2015 | | 48h | | 34/34 | 41/27 | 62.3 35-78 | SI0.2g+WM | | WM | 14d | ① | 2 |
| Wang 2016 | | 24h | | 41/40 | 48/33 | 60.1 42-80 | SI0.2g+WM | | WM | 14d | ①②⑥⑦⑧ | 2 |
| Zhang 2015 | | 72h | | 40/40 | 43/37 | 61.8 | SI0.2g +WM | | WM | 14d | ②⑦ | 3 |
| Zhang 2010 | | 72h | | 60/60 | 67/53 | 65.1 40-80 | SI0.2g+WM | | WM | 14d | ②④⑤⑥⑦ | 4 |
| Zhao 2015 | | 72h | | 80/80 | 95/65 | 58 48-79 | SI0.25g+WM | | WM | 15d | ①②⑧ | 5 |
| Zhou 2015 | | 72h | | 91/91 | 106/76 | 61.2 33-72 | SI0.2g+WM | | WM | 14d | ① | 2 |
| Zhu 2016 | | 72h | | 49/49 | 56/42 | 66.5 60-78 | SI0.2g+WM | | WM | 14d | ②③④⑤⑥ | 2 |
| Zhang 2015 | | 48h | | 35/35 | 42/28 | 57.7 37-79 | DSSA0.1g+WM | | WM | 14d | ②③⑧ | 4 |
| Hao 2014 | | 24h | | 47/47 | 52/42 | 51.8 40-69 | DSSA0.3g+WM | | WM | 14d | ③⑦ | 2 |
| Li 2015 | | 96h | | 50/50 | 65/35 | 55.2 48-96 | DSSA0.13g+WM | | WM | 14d | ①②③④⑤⑧ | 2 |
| Xu 2015 | | 1w | | 53/53 | - | 30-75 | DSSA0.13g+WM | | WM | 14d | ②③ | 2 |
| Liu 2004 | | 72h | | 36/30 | 36/30 | 49-80 | GXN20ml+WM | | DS+WM | 14d | ① | 1 |
| Chen 2007 | | 72h | | 60/60 | 67/53 | 59.0 37-78 | DH20ml+WM | | FFDS20ml+WM | 14d | ①②④⑤⑥⑦⑧ | 2 |
| Chen 2008 | | 1w | | 36/36 | 42/30 | 40.2 32-45 | DH20ml+WM | | FFDS 20ml+WM | 15d | ①② | 2 |
| Huang 2012 | | 48h | | 90/90 | 96/84 | 69.8 | DH10ml+WM | | FFDS10ml+WM | 14d | ①⑦ | 4 |
| Jiang 2008 | | 72h | | 40/40 | 45/35 | 60 35-80 | DH20ml+WM | | FFDS16ml+WM | 14d | ①⑧ | 2 |
| Lei 2012 | | 72h | | 60/60 | 65/55 | 61.5 | DH30ml+WM | | FFDS20ml+WM | 14d | ①⑧ | 2 |
| Li 2012 | | 24h | | 36/36 | 50/22 | 62.2 51-76 | DH40ml+WM | | FFDS40ml+WM | 14d | ①② | 4 |
| Li 2009 | | 72h | | 86/86 | 110/62 | 68.5 43-87 | DH40ml+WM | | FFDS40ml+WM | 14d | ①②④⑤⑦⑧ | 2 |
| Liu 2007 | | 72h | | 70/70 | 83/57 | 67.6 40-72 | DH40ml+WM | | FFDS20ml+WM | 14d | ①②④⑤⑥⑦⑧ | 2 |
| Ma 2007 | | 72h | | 66/66 | 74/58 | 59 37-78 | DH40ml+WM | | FFDS20ml+WM | 14d | ①④⑤⑥⑦⑧ | 2 |
| Shi 2008 | | 72h | | 40/40 | 41/39 | 43-79 | DH30ml+WM | | FFDS20ml+WM | 21d | ①②⑥⑦ | 2 |
| Shi 2009 | | 72h | | 45/45 | 49/41 | 64.9 43-79 | DH20ml+WM | | FFDS20ml+WM | 30d | ①② | 2 |
| Zhang 2009 | | 72h | | 54/56 | 65/45 | 64.0 40-80 | DH20ml+WM | | FFDS20ml+WM | 14d | ①⑧ | 2 |
| Tao 2012 | | 72h | | 76/76 | - | 43-81 | DH20ml+WM | | FFDS+WM | 14d | ①②④⑤⑥⑦ | 2 |
| Wang 2010 | | 72h | | 40/40 | 45/35 | 61.7 40-73 | DH20ml+WM | | FFDS20ml+WM | 15d | ①② | 2 |
| Zhang 2008 | | 72h | | 43/43 | 43/43 | 55.3 40-79 | DH20ml+WM | | FFDS20ml+WM | 14d | ①② | 3 |
| Zhu 2008 | | 1w | | 40/40 | 53/27 | 35-85 | DH30ml+WM | | FFDS20ml+WM | 14d | ①②④⑤⑥ | 2 |
| Yuan 2015 | | 8h | | 70/70 | 79/61 | 64-78 | DSCXQ15ml+WM | | DS20ml+WM | 15d | ①②③⑥⑦ | 2 |
| Feng 2015 | | 72h | | 50/50 | 52/48 | 48-78 | DSCXQ10ml+WM | | FFDS20ml+WM | 14d | ①②③ | 2 |
| Yang 2010 | | 72h | | 26/24 | 32/18 | 66.7 | DSCXQ30ml+WM | | FFDS20ml+WM | 14d | ① | 2 |
| Ma 2011 | | 48h | | 90/90 | 102/78 | 59.3 42-76 | DSCXQ10ml+WM | | FFDS20ml+WM | 14d | ①⑧ | 2 |
| Chen 2013 | | 72h | | 56/57 | 64/49 | 60.3 45-81 | DSCXQ10ml+WM | | FFDS20ml+WM | 14d | ①② | 2 |
| Hao 2011 | | 72h | | 64/58 | 71/51 | 50-75 | DSCXQ10ml+WM | | FFDS20ml+WM | 14d | ①②④⑤⑦⑧ | 2 |
| Yan 2016 | | 6-48h | | 30/30 | 31/29 | 52.7 32-68 | DSCXQ10ml+WM | | FFDS20ml+WM | 14d | ①②③ | 2 |
| Yang 2007 | | 24h | | 48/36 | 50/34 | 53.5 39-75 | DSCXQ10ml+WM | | FFDS20ml+WM | 30d | ①②⑧ | 2 |
| Ye 2007 | | 48h-1w | | 45/45 | 46/44 | 63.8 | STS6ml+WM | | FFDS20ml+WM | 15d | ①⑧ | 2 |
| Yang 2012 | | 24h | | 60/60 | 63/57 | 67.6 53-88 | DH20ml+WM | | DS20ml+WM | 14d | ①⑥⑧ | 2 |
| Lian 2011 | | 24h | | 50/50 | 71/29 | 42-72 | DH20ml+WM | | DS20ml+WM | 14d | ①②⑧ | 2 |
| Lu 2009 | | 72h | | 40/40 | 47/33 | - | DH30ml+WM | | DS20ml+WM | 15d | ①②⑥⑦⑧ | 2 |
| Liu 2012 | | 72h | | 20/20 | 23/17 | - | DH20ml+WM | | DS20ml+WM | 14d | ①④⑤⑥⑦⑧ | 2 |
| Li 2013 | | 6h-2w | | 30/30 | 27/33 | 67.1 48-82 | DH30ml+WM | | DS6ml+WM | 14d | ①②③ | 2 |
| Du 2013 | | 2-110h | | 30/30 | 38/22 | - | DH40ml+WM | | DS+WM | 28d | ① | 2 |

Note: C=control group, d= day, DH=Danhong injection, DS=Danshen injection, DSCXQ=Danshenchuanxiongqin injection, DSSA=Danshen Salvianolic Acids injection, E= experimental group, F= female, FFDS=Fufang Danshen injection, GXN=Guanxinning injection, M= male, SI=Salvianolate injection, STS=Sodium Tanshinone ⅡA Sulfonate injection, WM=western medicien, ①=clinical effective rate, ②=neurological impairment, ③=activities of daily living function, ④=whole blood high shear viscosity, ⑤=whole blood low shear viscosity, ⑥=plasma viscosity, ⑦=fibrinogen, ⑧=ADRs/ADEs.

Reference

[1] Li ZK. Clinical observation of Salvia miltiorrhiza injection in the treatment of acute ischemic stroke. Chin J Prim Pharm 2007;14:1542-3.

[2] Shen AR, Shi XD, Li XF, et al. Effect of low molecular heparin combined with Danshen Injection on hemorheology in patients with acute cerebral infarction. Chin J Hemorheol 2007;17: 549-51.

[3] Xia XY. Clinical observation of Salvia miltiorrhiza injection combined with edaravone in the treatment of acute cerebral infarction. Chin J Clin Ration Drug Use 2016;9:63-4.

[4] Yu HY, Kang LM, Wang YY, et al. Effect of Danshen injection on thromboxane A2 and prostacyclin in patients with acute cerebral infarction. Chin J Postgr Med 2010;33:30-1.

[5] Hao WQ, Li XL, Cheng ZX, et al. The influence of compound root of red-rooted salvia on hematological parameters in the patients with acute cerebral infarction. Clin J Med Offic 2005;33:433-5.

[6] Li AP, Sui WL. Effect of compound Danshen injection on fibrinolytic system in patients with acute cerebral infarction. J New Chin Med 2014;46: 35-7.

[7] Liang JR, HuangFu B. Efficacy of compound Danshen injection combined with edaravone on patients with acute cerebral infarction and its effect on copeptin, NT-proBNP and Hcy levels. Chin J Biochem Pharm 2016;36:144-6.

[8] Lv GP. Curative effect observation compound Danshen injection on acute cerebral infarction. Public Med Forum Mag 2014;18:4779-80.

[9] Shi JQ, Yang CL, Wu GC. Effect of compound Danshen and batroxobin injection on neurologic impairment and activity of daily living of elderly people with progressive cerebral infarction. Youjiang Med J 2012;40:616-8.

[10] Zhang X, Hu S, Li ZJ. Effect of compound Danshen injection on inflammatory factors in patients with acute cerebral infarction. Chin Mod Doct 2007;15:6-8.

[11] Zheng ZX, Gao Q, Chen H. Curative effect observation of cinepazide maleate combined with compound Danshen injection in treatment of acute cerebral infarction. J Guangdong Med Coll 2014;32:519-20.

[12] An YP, Ren CS. The clinical observation of Danhong injection edaravone in treatment of acute cerebral infarction. World Latest Med Inf 2015;15:41-75.

[13] Chen F, Xu ZR. Clinical observation on 134 cases of cerebral infarction treated with Danhong injection. Chin Med Mod Dis Educ China 2013;11:24-5.

[14] Cheng L. Clinical study of Danhong injection on high sensitivity C reactive protein in patients with acute massive cerebral infarction. Chin Foreign Med Res 2010;8:69-70.

[15] Cui ZW. Danhong injection combined with edaravone injection in the treatment for 40 cases of cerebral infarction. West J Tradi Chin Med 2014;27:85-6.

[16] Du GS. Clinical analysis on 57 cases of acute cerebral infarction treated with Danhong injection. World Health Dig Med Periodical 2011;8:422-3.

[17] Gu YX, Li P. Clinical observation of sodium ozagrel combined with Danhong injection in the treatment of acute cerebral infarction. China Pract Med 2012;7:176-8.

[18] Gu L, Wang JJ. The curative effect observation of Danhong injection combined with atorvastatin calcium in the treatment of lacunar cerebral infarction of the elderly. Int J Tradi Chin Med 2014;36:519-21.

[19] Han ZH. Effect of Dan Hong injection on acute cerebral infarction in patients with serum cytokine levels. J Tradit Chin Med Univ Hunan 2009;29:29-30.

[20] Hao JS. Clinical observation of Danhong injection in the treatment of acute cerebral infarction in 124 cases. China Foreign Med Treat 2008;27:84.

[21] Hao YP. Clinical observation of sodium ozagrel combined with Danhong injection in the treatment of acute cerebral infarction. Chin J Pract Nerv Dis 2013;16:70-1.

[22] Hu J, Jiang XP. Effect of Danhong injection on hemorheology of patients with acute cerebral infarction. China Pharm 2008;11:691-3.

[23] Jiang CP, Wu BH, Liu F, et al. Effects of Danhong injection on plasma levels of TNF-α and IL-6 and its clinical efficacy in patients with acute cerebral infarction. J China Pharm 2008;19:1900-2.

[24] Jiang XF. Danhong injection in the treatment of acute ischemic stroke: a report of 31 cases. Shaanxi J Tradi Chin Med 2011;32:1301-2.

[25] Li CS, Gao YH, Hu FF, et al. Clinical observation of Danhong injection combined with batroxobin injection for 60 cases of acute evolving cerebral infarction. World Chin Med 2010;5:243-5.

[26] Li GL, Zhao WL, Wang HQ, et al. Danhong injection effect on neurological function and serum Cys-C levels of patients with acute cerebral infarction. Liaoning J Tradit Chin Med 2015;42:2342-3.

[27] Li H, Zhang L, Yang XP. The clinical observation of Danhong injection treating cerebral infarction. Chin J Clin Ration Drug Use 2012;5:5-6.

[28] Li HQ. Danhong injection for 40 cases of ischemic stroke. Chin Commun Doct 2012;14:153.

[29] Li HM. Clinical observation on Danhong injection in treating acute cerebral infarction. China J Chin Med 2011;26:1234-5.

[30] Li J, Xu S. Influence of Danhong injection on the oxidative stress and the serum cytokine level in acute cerebral infarction patients. World Clin Drug 2015;3:185-8.

[31] Li M, Li Q, Zhang M, et al. Effect of Danhong injection on levels of serum s100 b protein and neuron_specific enolase in patients with acute cerebral infarction. Her Med 2014;33:1596-9.

[32] Li X. Danhong injection in the treatment of type 2 diabetes patients with cerebral infarction. Pract J Cardiac Cereb Pneum Vasc Dis 2010;18:1436-7.

[33] Liang ZH, Zhang HL, Qi DB, et al. Effect of Danhong injection on serum inflammatory factors in patients with acute ischemic stroke. J Emerg Syndromes Tradit Chin Med 2014;23:2115-6.

[34] Liu JM. Efficacy analysis of Danhong injection treatment of acute cerebral infarction. J Henan Univ Sci Technol ( Med Sci) 2013;31:30-1.

[35] Liu XW, Yang L, Yang R. Clinical observation of Danhong injection combined with aspirin in treatment of cerebral infarction. Chin J Misdiagn 2008;8:33-4.

[36] Liu YP, Yu ZG, Wu H. Danhong injection on acute cerebral infarction C-reactive protein and clinical effects. Pract J Cardiac Cereb Pneum Vasc Dis 2010;18:1433-4.

[37] Luo YF, Xu DD, Xu XP. Danhong injection combined with ozagrel for 90 cases of acute cerebral infarction. Chin J Prim Med Pharm 2012;19:3136-7.

[38] Ma J, Xu NW, Wu HH, et al. Effect of Danhong injection on nerve function and hemorheology for patients with acute ischemic stroke. Chin J Exp Tradit Med Form 2015;21:204-7.

[39] Mao JJ. Clinical observation on the treatment of 58 cases of acute cerebral infarction with Danhong injection combined with Edaravone injection. Int J Tridi Chin Med 2010;32:507-8.

[40] Mu PY, Hui LL, Song HY, et al. Integrative Chinese and Western medicine for treating 30 cases of senile cerebral infarction. Henan Tradit Chin Med 2015;35:1535-6.

[41] Ni QM. Clinical observation of Danhong injection in the treatment of acute ischemic stroke. J Pract Tradit Chin Med 2015;31:749-50.

[42] Qi LS, Jia CW, Xie Z. Effect of Danhong Injection on plasma endothelin and nitric oxide levels in patients with cerebral infarction. Chin J Integr Med Cardio/ Cerebrovasc Dis 2013;11:1477-8.

[43] Qiao B. Clinical observation of Danhong injection in the treatment of 60 cases of acute cerebral infarction. China Foreign Med Treat 2010;29:116.

[44] Shen HJ. Clinical observation of Danhong injection combined with edaravone in the treatment of cerebral infarction. Guide China Med 2012;10:580-1.

[45] Su XC, Luo SG, Long JY. Danhong injection in the treatment of 60 cases of cerebral infarction. Guangxi Med J 2012;34:1543-5.

[46] Tang KQ, Lu DL, Qiang H. Effect of Danhong injection on hypersensitivity C reactive protein in patients with acute cerebral infarction. Chongqing Med J 2008;37:2876-7.

[47] Tao WB. Application of Danhong Injection in Treating Acute Cerebral Infarction. Heilongjiang Med J 2011;35:602-4.

[48] Tao YC, Hu YX, Ding HZ. The effects of batroxobin plus Danhong injection in the treatment of progressive cerebral infarction. People’s Mil Surg 2012;55:35-6.

[49] Tian J, Chang H, Cui Y, et al. Clinical observation of Danhong injection in the treatment of acute cerebral infarction. China Pract Med 2010;5:161-2.

[50] Wang DF. Clinical observation of edaravone combined with Danhong injection in the treatment of cerebral infarction. China Health Nutr 2015;12:309-10.

[51] Wang LT, Zhang WW, Huang YH, et al. The Effects of Danhong Injectionon neurological function and serum CRP in patients with acute cerebral infarction. Pract J Cardiac Cereb Pneum Vasc Dis 2008;16:27-8.

[52] Wang TH. Effect of Danhong Injection on serum inflammatory factors in patients with acute cerebral infarction. Chin J Pract Nerv Dis 2011;14:44-6.

[53] Wang XJ, Guo JQ, Xu SZ, et al. Effect of Danhong Injection on compensatory circulation in patients with acute cerebral infarction. Chin Tradit Pat Med 2015;37:1624-6.

[54] Wang XR. Clinical observation of Danhong injection in the treatment of acute cerebral infarction. China Pract Med 2008;3:57-8.

[55] Wu H. Clinical observation of Danhong injection on acute cerebral infarction patients. Med Inno China 2012;9:38-9.

[56] Wu XQ, Huang HF. Effect of Danhong Injection on platelet leukocyte aggregates and high sensitivity C reactive protein in patients with acute cerebral infarction. Zhejiang J Tradit Chin Med 2013;48:648-9.

[57] Wu XH. Clinical observation of sodium ozagrel combined with Danhong injection in the treatment of acute cerebral infarction. Yiayao Qianyan 2014;4:187-8.

[58] Xie SG. Danhong injection in the treatment of 30 cases of cerebral infarction. Yunnan J Tradit Chin Med Mater Med 2008;29:26.

[59] Xu J. Efficacy analysis of edaravone combined with Danhong injection in the treatment of elderly patients with acute cerebral infarction. China Contin Med Edu 2014;6:71-2.

[60] Xuan ZH. Danhong injection in the treatment of acute atherosclerotic cerebral infarction: a report of 60 cases. Zhejiang J Tradit Chin Med 2011;46:309.

[61] Yan YP. Effect of Danhong injection combined with sodium ozagrel on serum homocysteine level in patients with cerebral infarction. Proc Clin Med 2013;22:22-4.

[62] Yang LS. Effect of Danhong Injection on plasma visfatin in patients with acute ischemic stroke. Chin J Pract Nerv Dis 2015;18:46-9.

[63] Yin LL. Danhong injection combined with sodium ozagrel injection in the treatment of 40 cases of acute cerebral infarction. Zhejiang J Tradit Chin Med 2010;45:778.

[64] Zhang HY. Danhong injection combined with sodium ozagrel injection in the treatment of acute cerebral infarction: a report of 64 cases. Zhejiang J Tradit Chin Med 2012;47:327.

[65] Zhang LH, Fu DM, Ji W. Clinical observation of Danhong injection in the treatment of acute cerebral infarction. Public Health Work China 2008;16:644.

[66] Zhang M. Effects of Danhong injection in patients with acute cerebral infarction. Hubei Univ Tradit Chin Med 2015.

[67] Zhang QM. Clinical observation of Danhong injection combined with sodium ozagrel in the treatment of acute cerebral infarction. Chin J Misdiagn 2011;11:6853.

[68]Zhang SG. Clinical observation on 40 cases of acute cerebral infarction treated with combination of traditional Chinese medicine and Western Medicine. Henan Tradit Chin Med 2013;33:1528-9.

[69] Zhang XT. Curative effect observation of Danhong injection combined with edaravone injection in the treatment of 90 cerebral infarction cases. China Pract Med 2015;10:36-7.

[70] Zhang YB, Wang J, Li S, et al. Danhong injection combined with edaravone in the treatment of elderly patients with acute cerebral infarction. Acta Guangxi Med Univ 2011;28:447-8.

[71] Zhang ZP. Clinical observation of Danhong injection in the treatment of acute cerebral infarction. Pract J Cardiac Cereb Pneum Vasc Dis 2010;18:39.

[72] Zhong CY. Efficacy analysis of edaravone combined with Danhong injection in the treatment of acute cerebral infarction. China Med Pharm 2011;1:66.

[73] Zhou YJ. Clinical observation of Danhong injection in the treatment of acute cerebral infarction. Jilin Med J 2007;28:464-5.

[74] Fang HW, Huang XY, Mei ZZ, et al. Clinical observation of Colin Bay combined with Danhong injection on acute cerebral infarction in 48 cases. Chin J Ethnomed Ethnopharm 2013;22:54-5.

[75] Li FZ. Danhong injection combined with low molecular heparin calcium in the treatment of 68 cases of cerebral infarction. Natl Med Front China 2013;8:70-1.

[76] zhu JL. Observation of combination of Danhong injection and aspirine therapy in treating patients with cerebral infarction. J Liaoning Med Univ 2012;33:125-7.

[77] Guo Y. Analysis of curative effect by salviae miltiorrhizae ligustrazine injectioncombined withedaravone injection in the treatment of acute cerebral infarction. Chin J Mod Drug Appl 2015;9:20-1.

[78] Lan Y, Xiao JX, Zheng TY, Liu ZX. Effect of Danshenchuanxiongqin injection on lysophosphatidic acid and P selectin in patients with acute cerebral infarction. Mod J Integr Tradit Chin West Med 2015;24:840-2.

[79] Li J, Cheng H. Clinical observation of deproteinated calf blood hydrolysate combined with Danshenchuanxiongqin in the treatment of acute cerebral infarction. Xinjiang J Tradit Chin Med

Pharm 2014;32:17-8.

[80] Ling JY, Liu Q, Wu JS, Shen L. Effects of Danshenchuanxiongqin injection on pac-1 and cd62p in patients with acute ischemic stroke. Res Integrated Tradit West Med 2013;5:60-2.

[81] Liu CL. Effect of Danshenchuanxiongqin injection on hemodynamics in patients with acute cerebral infarction. Med Inno China 2011;8:88-9.

[82] Liu H, Tong YY, Han M, et al. Therapeutic effects of Danshenchuanxiongqin injection with brain glycosides peptide injection on patients with acute ischemic stroke. For All Health 2015;9:156.

[83] Liu M. Efficacy and safety of edaravone alone and in combination with Danshenchuanxiongqin in the treatment of acute cerebral infarction. Mod J Integr Tradit Chin West Med 2014;23:2105-7.

[84] Wan J, Yang XW, Zhu LL. Effect of Danshenchuanxiongqin injection on neurologic impairment and activity of daily living in patients with acute cerebral infarction. Lishizhen Med Mater Med Res 2015;26:659-60.

[85] Wang MP, Luo WC, Yang JQ. Clinical observation of edaravone combined with Danshenchuanxiongqin in the treatment of acute cerebral infarction. Mod J Integr Tradit Chin West Med 2015;24:1533-5.

[86] Wang TQ. Integrated traditional Chinese and western medicine therapy in 30 patients with acute cerebral infarction. China Mod Med 2012;19:160-1.

[87] Yang ZY, Zhang HL, Luo XH. Clinical observation on 31 cases of acute cerebral infarction treated with Danshenchuanxiongqin combined with brain protein hydrolysate. Yunnan J Tradit Chin Med Mater Med 2010;31:21-2.

[88] Ye ZF, Cheng YZ. Clinical observation on 40 cases of acute cerebral infarction treated with Danshenchuanxiongqin combined with atorvastatin. J Med Theory Pract 2013;26:2155-6.

[89] Zhang Y, Hou J, Hu Y, Mu ZB, Hou YM. Clinical observation of Danshenchuanxiongqin and Ligustrazine Hydrochloride Injection combined with edaravone in the treatment of acute cerebral infarction. Chin J Integr Med Cardio/ Cerebrovasc Dis 2012;10:168-9.

[90] Zhao KS, Fang DJ. Effect of Danshenchuanxiongqin injection on serum MMP-9 level in patients with acute cerebral infarction. Mod J Integr Tradit Chin West Med 2015;24:2542-4.

[91] Zhao SP. Clinical study on cerebral infarction treated with integrated traditional Chinese and Western medicine therapy. Guide China Med 2013;11:283-4.

[92] Hu HQ. Clinical observation on 40 cases of acute cerebral infarction treated with Danshenchuanxiongqin combined with atorvastatin. Diet Health 2015;1:64-5.

[93] Wang XM, Zhang ML. Effect of Danshenchuanxiongqin injection on lysophosphatidic acid, plasma endothelin, P-selectin in patients with acute cerebral infarction. Mod J Integr Tradit Chin West Med 2016;25:2118-20.

[94] Li CL, Zha XC, Sun YH. Effect of Danshenchuanxiongqin injection on serum VCAM-1, ICAM-1, ET-1 and NO in elderly patients with acute cerebral infraction. Mod J Integr Tradit Chin West Med 2016;25:2088-90.

[95] Gong JF, An LH. Clinical analysis of Tanshinone IIA Sulfonate injection in the treatment of cerebral infarction. Jilin Med J 2009;30:431-2.

[96] Long Y. Observation of therapeutic effect of sodium ozagrel combined sodium tanshinone Ⅱ A silate in treating acute cerebral infarction. China Mod Med 2010;17:51-2.

[97] Kang HB. Clinical study on the tanshinone IIA sodium sulfonate injection in the treatment of patients with acute cerebral infarction. World Latest Med Inf 2015;15:105-6.

[98] Li LZ, Liu JJ, Zhang M, Guo LH. Effects of sodium tanshinone IIA sulfonate combined with edaravone on focal cerebral infarction blood flow and blood rheology. J Liaoning Univ Tradit Chin Med 2016;18:182-4.

[99] Ma XD, Zhan PJ, Xi ZH. Clinical observation of tanshinone II A sulfonate combined with edaravone in the treatment of acute cerebral infarction. J Emerg Syndromes Tradit Chin Med 2012;21:291-2.

[100] Tan WH, Wei R. Clinical observation of early application of tanshinone II A sulfonate injection in the treatment of acute cerebral infarction. J Guiyang Coll Tradit Chin Med 2008;30:29-31.

[101] Xu SM. Clinical study of clopidogrel combined with tanshinone II A sulfonate injection in the treatment of acute cerebral infarction. Chin J Clin Res 2015;28:310-2.

[102] Yang J. Clinical observation of tanshinone II A sulfonate in the treatment of 60 patients with cerebral infarction. Guide China Med 2013;11:117-8.

[103] Zhang HT. Clinical observation of tanshinone II A sulfonate in the treatment of acute cerebral infarction. Chin J Pract Nerv Dis 2016;19:97-9.

[104] Zhang X, Zhang LY. Clinical study of Tanshinone Ⅱ A for treatment on acute cerebral infarction. J Clin Neurol 2008;21:458-9.

[105] Zhou J, Yu BL. Clinical observation of tanshinone II A sulfonate in the treatment of acute cerebral infarction. Sichuan J Tradit Chin Med 2014;32:172-3.

[106] Shan XY. The effect of Salvianolate injection on the cognitive dysfunction after acute stroke. Chin J Pract Nerv Dis 2016;19:108-9.

[107] Duan FR. Clinical observation of Salvianolate injection combined with edaravone in the treatment of acute cerebral infarction. Med Inf 2013;26:352-3.

[108] Hao SJ, An HJ. Observation of the curative effect of Salvianolate injection in the treatment of acute cerebral infarction. Chin J Integr Med Cardio/ Cerebrovasc Dis 2012;10:1076-7.

[109] Ji PZ, Luo ZQ, Wang CG. Effectiveness of depside salt from Salvia miltiorrhiza in the treatment of acute ischemic stroke. Pharm Care Res 2010;10:4.

[110] Li QL, Yao HY, Zhong GX, et al. Effectiveness of depside salt from Salvia miltiorrhiza in the treatment of acute ischemic stroke. J North Pharm 2016;13:58-9.

[111] Mi YX. The protective effect of salvianolate on vascular endothelial function in patients with acute cerebral infarction. Chin J Pract Nerv Dis 2013;16:9-11.

[112] Nie XD, Wu XM. Clinical observation of Salvianolate injection in the treatment of acute cerebral infarction. Summit Forum of Clinical Acute Severe Experience Communication 2015.

[113] She JH, Lin DW. Clinical study of edaravone combined with Salvianolate injection in the treatment of acute cerebral infarction. Beijing Med J 2014;36:512-3.

[114] Tao Z, Cao BZ, Wang SC, et al. Effects of Salvianolate injection on serum hs-CRP, hemorheology and neurological function in elderly patients with acute ischemic stroke. Chin J Pract Nerv Dis 2015;18:29-31.

[115] Wang F, Yang Q. Effectiveness of depside salt from Salvia miltiorrhiza in the treatment of acute ischemic stroke. Summit Forum of Clinical Acute Severe Experience Communication 2015.

[116] Wang Q, Zhang YP, Zhan Y. Clinical observation of Salvianolate injection in the treatment of acute cerebral infarction. Chin J Clin Ration Drug Use 2016;9:29-30.

[117] Zhang XW. The effect of Salvianolate injection on fibrinogen and C- reactive protein in the treatment of acute cerebral infarction. Zhejiang J Tradit Chin Med 2015;50:544.

[118] Zhang Z, Yin ZL, Liu YJ. Clinical observation of Salvianolate injection in the treatment of patients with acute cerebral infarction. J Hunan Norm Univ (Med Sci) 2010;3:65-7.

[119] Zhao Y. Influence of Salvianolic acid in acute cerebral infarction. Health Way 2015;2:63-4.

[120] Zhou BZ. Influence of Salvianolic acid in acute ischemic stroke. Henan Med Res 2015;24:120-1.

[121] Zhu JZ, Yang B, Sun Y, et al. Influence of salvia miltiorrhiza polyphenols keto acid salt on the serum hs-crp, hemorheology and nerve function of elderly patients with acute ischemic stroke. Prog Mod Biomed 2016;16:2500-2.

[122] Zhang F, Qiu J, Zhang LM, et al. Clinical study on the effect of salvianolate on motor and cognitive function of patients with acute cerebral infarction. Chin J Clin Healthc 2015;18:232-4.

[123] Hao YC, Su J. Effect of Danshen polyphenolic acid on hemorheology and neurological function in patients with cerebral infarction. Chin J Pract Nerv Dis 2014;17:67-8.

[124] Li HJ. Effects of Zhesheyong Danshenduofensuan injection on acute cerebral infarction . Yanan Univ 2015.

[125] Xu W, Wang CX, Han H, et al. Clinical study on the treatment of Salvianolic Acid for mild to moderate cerebral infarction. J Clin Rat Drug Use 2015; 8:14-5.

[126] Liu YF. Effects of Guanxinning injection on acute cerebral infarction . Lishizhen Med Mater Med Res 2004;9:644.

[127] Chen YB, Wu HP, Bao XY. Clinical observation of Danhong injection in the treatment of lacunar infarction. Zhejiang J Integr Tradit Chin West Med 2007;17:749-50.

[128] Chen ZX, Li XJ, Zhao YH. Danhong Injection for treatment of young patients with acute cerebral infarction. Chin J Integr Med Cardio 2008;6:1036-7.

[129] Huang AJ. Effects of Danhong injection on senior citizen with acute cerebral infarction . China Mod Med 2012;19:67-8.

[130] Jiang HL, Wang YK, Li W. Observation of Danhong injection in the treatment of cerebral infarction. China Healthc Innovat 2008;3:57.

[131] Lei JF. Effects of Danhong injection on acute cerebral infarction. Chin foreign health abstr 2012;9:243-4.

[132] Li JQ. Effects of Danhong injection on acute cerebral infarction and its effects on hypersensitive c-reactive protein and d-dimer. Chin J Integr Med Cardio 2012;10:305-6.

[133] Li ZF, He Y, Peng SX, et al. The effect of Danhong injection on hemorheology and hemodynamics of patients with acute cerebral infarction and it's clinical efficacy. China Pract Med 2009;4:16-8.

[134] Liu WS, Wang J. Observation of Beitong injection in the treatment of acute cerebral infarction. Chin J Pract Nerv Dis 2007;10:60-1.

[135] Ma XD, Xu JH, Zhan PJ. Clinical observation of Danhong injection in the treatment of acute cerebral infarction. Zhejiang J Integr Tradit Chin West Med2007;17:136-7.

[136] Shi JP, Zhang Y. Clinical observation of Danhong injection in treating 40 cases of cerebral infarction. Shaanxi J Tradit Chin Med 2008;29:1499-500

[137] Shi XY. Clinical observation of Danhong injection in treating 45 cases of cerebral infarction. Pract J Cardiac Cereb Pneum Vasc Dis 2009;17:689.

[138] Sun HQ, Shi GL. Research on Danhong injection in the treatment of acute cerebral infarction. Chin J Clin Ration Drug Use 2009;2:1-2.

[139] Tao YH, Zhao LY. Observation of Danhong injection in the treatment of acute cerebral infarction. Med Front 2012;32:195-6.

[140] Wang ZF, Huo GQ. Clinical observation of Danshenchuanxiongqin injection in treating 80 cases of cerebral infarction. China Healthc Innovat 2010;5:16.

[141] Zhang JB, Zhang LL, Wang SF, et al. Clinical research of danhong injection on cerebral hemodynamics in patients with acute cerebral infarction. China Practical Medical 2008;3:14-6.

[142] Zhu HJ. Clinical observation of Danhong injection in treating 56 cases of acute cerebral infarction. Zhejiang J Tradit Chin Med 2008;43:556.

[143] Yuan W. Evaluation of N FDS score and Barthel index of senile acute cerebral infarction patients with the treatment of danshen ligustrazine injection. China Pract Med 2015;18:24-5.

[144] Feng YX. The effect of Salvia Ligustrazine injection on the neurological function deficiency and barthel index in patients with acute cerebral infarction. Chin Med Mod Dis Educ China 2015;13:15-6.

[145] Yang SJ. Observation of Danshenchuanxiongqin injection in the treatment of acute cerebral infarction. China Pract Med 2010;5:134-5.

[146] Ma CL. Observation of Danshenchuanxiongqin injection and Edaravone Injection in treating 90 cases of acute cerebral infarction. A seminar on the integration of neuromedicine in Chinese and western medicine 2011.

[147] Chen HB, Zhang BY. Clinical observation of Danshenchuanxiongqin injection in treating 56 cases of acute cerebral infarction. J Clin Med Pract 2013;17:96-7.

[148] Hao YH, Qi JH, Jia LL. Clinical observation of Danshenchuanxiongqin injection in the treatment of acute cerebral infarction. Qingdao Med J 2011;42:364-6.

[149] Yan SJ. Observation of Danshenchuanxiongqin injection in treating acute cerebral infarction. J New Chin Med 2016;48:19-21.

[150] Yang HJ. Clinical efficacy of Xuetong injection in the treatment of acute cerebral infarction in elderly patients. J Pract Med Tech 2007;14:2157.

[151] Ye XS, Xu YG, Zhang ZW, et al. Clinical observation of Sodium Tanshinone ⅡA Sulfonate injection in treating acute cerebral infarction. Chin Med Rep 2007;4:82.

[152] Yang LJ. Clinical efficacy of Danhong injection in the treatment of acute cerebral infarction in elderly patients. Pract J Cardiac Cereb Pneum Vasc Dis 2012;20:2017–8

[153] Lian CL, Lu AN, Lian X, et al. Clinical observation of Danhong injection in treating 50 cases of acute cerebral infarction. Chin Commun Doct 2011;13:197–8.

[154] Lu JY, Nan H, Tian LP, et al. Observation on therapeutic effect of Danhong injection on acute cerebral infarction. Chin Commun Doct 2009;11:118.

[155] Liu LQ. Clinical observation of Danhong injection on serum highsensitivity C-reactive protein in patients with acute cerebral infarction. Jilin J Tradit Chin Med 2012;32:370–1.

[156] Li WJ, Xie X, Huang WQ, et al. Clinical analysis of changes on serum Hcy levels in patients with cerebral infarction after using Danhong injection. Prev Treat Cardio Cereb Vasc Dis 2013;13:26–8.

[157] Du L, Wang XH. Observe the Danhong injection after treatment in patients with cerebral infarction. Health World 2013;34:206
